# Machine Learning Identifies Complicated Sepsis Course and Subsequent Mortality Based on 20 Genes in Peripheral Blood Immune Cells at 24 H Post-ICU Admission

**DOI:** 10.3389/fimmu.2021.592303

**Published:** 2021-02-22

**Authors:** Shayantan Banerjee, Akram Mohammed, Hector R. Wong, Nades Palaniyar, Rishikesan Kamaleswaran

**Affiliations:** ^1^Department of Pediatrics, University of Tennessee Health Science Center, Memphis, TN, United States; ^2^Department of Biotechnology, Indian Institute of Technology Madras, Chennai, India; ^3^Division of Critical Care Medicine, Cincinnati Children's Hospital Medical Center, Cincinnati, OH, United States; ^4^Translational Medicine, Peter Gilgan Center for Research and Learning, The Hospital for Sick Children, Toronto, ON, Canada; ^5^Department of Biomedical Informatics, Department of Emergency Medicine, Emory University School of Medicine, Atlanta, GA, United States; ^6^Department of Biomedical Engineering, Georgia Institute of Technology, Atlanta, GA, United States

**Keywords:** sepsis, complicated course, critical care, machine learning, transcriptomics, biomarkers

## Abstract

A complicated clinical course for critically ill patients admitted to the intensive care unit (ICU) usually includes multiorgan dysfunction and subsequent death. Owing to the heterogeneity, complexity, and unpredictability of the disease progression, ICU patient care is challenging. Identifying the predictors of complicated courses and subsequent mortality at the early stages of the disease and recognizing the trajectory of the disease from the vast array of longitudinal quantitative clinical data is difficult. Therefore, we attempted to perform a meta-analysis of previously published gene expression datasets to identify novel early biomarkers and train the artificial intelligence systems to recognize the disease trajectories and subsequent clinical outcomes. Using the gene expression profile of peripheral blood cells obtained within 24 h of pediatric ICU (PICU) admission and numerous clinical data from 228 septic patients from pediatric ICU, we identified 20 differentially expressed genes predictive of complicated course outcomes and developed a new machine learning model. After 5-fold cross-validation with 10 iterations, the overall mean area under the curve reached 0.82. Using a subset of the same set of genes, we further achieved an overall area under the curve of 0.72, 0.96, 0.83, and 0.82, respectively, on four independent external validation sets. This model was highly effective in identifying the clinical trajectories of the patients and mortality. Artificial intelligence systems identified eight out of twenty novel genetic markers (*SDC4, CLEC5A, TCN1, MS4A3, HCAR3, OLAH, PLCB1*, and *NLRP1*) that help predict sepsis severity or mortality. While these genes have been previously associated with sepsis mortality, in this work, we show that these genes are also implicated in complex disease courses, even among survivors. The discovery of eight novel genetic biomarkers related to the overactive innate immune system, including neutrophil function, and a new predictive machine learning method provides options to effectively recognize sepsis trajectories, modify real-time treatment options, improve prognosis, and patient survival.

## Introduction

Critically ill patients are admitted to the Intensive Care Unit (ICU) for complex and dynamic care, preserving organ function and improving outcomes in otherwise dire situations. Among patients with complicated disease courses, septic patients represent a significant component (1). Sepsis is a life-threatening organ dysfunction caused by the overactive immune response to bacterial infection, often of pulmonary origin (2). Sepsis may have contributed to 20% of all global deaths in 2019 (3). Sepsis consists of a heterogeneous mix of phenotypes (4, 5), various degrees of disease complexities, and trajectories leading to recovery or death (6, 7). Different strategies have been pursued predicting deterioration (8–10) and managing patients with sepsis in critical care units (11) using physiological, clinical, and biomarker parameters. However, due to the heterogeneous nature of patients presenting to the ICU and the diverse disease course that follows, it has been difficult to identify generalized models of disease (12).

Statistical and machine learning methods have been developed to successfully utilize the multi-omics data for biomarker discovery for predicting survival from sepsis (13). Wong et al. (14) identified 12 biomarkers collectively called the Pediatric Sepsis Biomarker Risk Model (PERSEVERE) class genes. Further analyses resulted in the identification of 18 additional genes consisting of the PERSEVERE XP set (15). Mohammed et al. (16) identified 53 differentially expressed genes, involved mostly in immune response and chemokine activity, from expression data collected from patients admitted to the pediatric ICU (PICU) within 24 h of admission. Sweeney et al. (17) analyzed the results obtained from three independent scientific groups that developed mortality prediction models and identified additional subgroups of genes. While much has been studied about the risk for mortality, there is a dearth of machine learning approaches to predict disease trajectory, including complicated disease courses and poor clinical outcomes (18). Early identification of disease trajectory, including complicated disease courses, defined as persistence of 2 or more organ failures by day 7 or death by day 28, can aid in clinical management and targeted therapies to manage severe outcomes. Hence, there is a need to identify these biomarkers and build novel machine learning models to identify complex disease courses from plasma samples collected close to the time of ICU admission.

In this work, we performed a meta-analysis of previously published peripheral blood cell gene expression data (sampled within 24 h of sepsis onset; 228 pediatric ICU patients) and analyzed them using multiple statistical and machine learning methods to identify novel markers of sepsis disease trajectory. We found 20 highly stable genes that predict disease complexity with an average derivation AUROC of 0.82 and validation AUROCs of 0.72, 0.96, 0.83, and 0.82 within critically ill children, using peripheral blood collected within 24 h of ICU admission. We validated these variables by calculating their overlap with the well-established sepsis mortality predicting genes, conducting the functional gene-set enrichment and pathway analyses, and testing them on four external validation datasets. Earlier identification of disease complexity can inform care management and targeted therapy. Therefore, the 20 gene candidates identified by our rigorous approach can be used to identify, early in their ICU stay, patients who may ultimately develop significant organ dysfunction and complex care management.

## Materials and Methods

### Data Collection

The pediatric sepsis dataset GSE66099 (19) downloaded from the NCBI Gene Expression Omnibus (GEO) repository, contains the gene expression profiles extracted from the peripheral blood samples of patients who were admitted to the PICU during the first 24 h of admission. The microarray dataset was obtained from the Affymetrix GPL570 platform, which was submitted by Wong et al. on February 19, 2015, and last updated on March 25, 2019. We considered a complicated course as the primary outcome variable. This was defined as death by 28 days or persistence ≥2 organ failures at day 7 of septic shock (20). This dataset was used to train our model and derive the top gene variables.

Due to the lack of available external data that encodes for complicated courses as defined in our derivation cohort, we used a collection of four microarray gene expression datasets as the closest surrogate to validate our list of biomarkers. The first dataset, GSE54514 (21), based on an adult cohort, provided whole blood samples collected up to 5 days after ICU admission. In this cohort, we defined the complicated presentation as patients with APACHE II scores ≥25 observed within 24 h of admission to the ICU. The second dataset, E-MEXP-3850 (22) was used to study the temporal evolution of sepsis by collecting whole blood samples at six different time points from five critically ill children admitted to the PICU with meningococcal sepsis and sepsis-induced multiple organ failure. Barring one sample from patient four that was degraded and not used for microarray analysis, there were a total of 29 distinct time-course based gene expression measurements included in this study. We considered 28-day mortality as the primary outcome variable for this cohort. The third dataset, E-MEXP-3567 (23) was used to discover biomarkers of severe bacterial infection using transcriptomic data collected from whole blood samples of children suffering from either bacterial meningitis or bacterial pneumonia. The outcome variable that was chosen for this dataset was in-hospital mortality. The fourth dataset, GSE40586 (24), was used to study the pathways activated at the transcriptional level by extracting RNA from the whole blood samples of patients (infants, children, and adults) suffering from bacterial meningitis and following a complicated clinical course. The outcome variables used in our analysis included the complications observed by a neurologist during a patient's discharge from the hospital. The processed expression data, probe to gene identifiers, and the outcome labels for the datasets GSE54514, E-MEXP-3850, E-MEXP-3567, and GSE40586 were downloaded from (17). The gene expression data were extracted from whole blood samples and thus contain a mixture of red blood cells, white blood cells and platelets. However, RNA from these sources do not contribute to the expression of the immune-related genes expressed in the white blood cells.

All procedures performed in studies involving human participants were in accordance with the ethical standards of the institutional review boards of all participating institutions, and with the Declaration of Helsinki and its later amendments or comparable ethical standards.

### Normalization and Background Correction

Technical variations in the gene expression data were eliminated using the Affy package (25) from R. This also helped remove the background noise. The Robust Multi-Average parameter method using “gcrma” package (26) from R was used to normalize data and perform background correction. Surrogate Variable Analysis was performed to infer batch effects and other unwanted sources of variation in the data. The “sva” package (27) in R was used for this purpose. The results of the surrogate variable analysis or the inferred batch effects were regressed with the actual batch variable (or year of measurements in this case) using the lm () function (28). The resulting variation was removed using the Combat () from the same package, and the cleaned expression set object was used for further analysis. Boxplots displaying the expression values before and after normalization and the relationship between inferred and observed batch effects were plotted using the boxplot () from R (28).

### Probe to Gene Mapping, Identification of Differentially Expressed Genes

For the microarray datasets GSE66099, GSE54514, E-MEXP-3850, E-MEXP-3567, GSE40586, the Affymetrix probes were matched to gene symbols using the Affymetrix Human Genome U133 Plus 2.0 (hgu133plus2.db), Illumina Human HT12v4 annotation database (illuminaHumanv4.db), Affymetrix Human Gene 1.0 ST Array (HuGene-1_0-st), Affymetrix Human Genome HG-U133A (hgu133a.db), and Affymetrix Human Gene 1.0 ST Array (HuGene-1_0-st), respectively. In order to detect the expression values of genes with multiple probes, we averaged the expression for multiple probes that matched to the same gene. The limma package (29) from R was used to perform the differential gene expression analysis with a Benjamini-Hochberg correction (FDR cutoff = 0.1).

### Functional Enrichment Analysis

We used the clusterProfiler package (30) in R to find enriched biological processes (BP), cellular components (CC), and molecular function (MF) terms. A plot displaying the enriched terms was drawn using the enrichplot package (31) in R. The STRING analysis online tool (32) was used to find significantly enriched Reactome and KEGG pathways.

### Statistical Analysis

Affymetrix data download and gene mapping were done using the “affy” and the annotation package “hgu133plus2.db” package in R, respectively. A Fisher exact test was performed to determine the statistical significance among the functional enrichment terms. Benjamini Hochberg's multiple test correction methods were used to calculate the differentially expressed genes (DEGs). Heatmap for the DEGs was generated using the Heatmap () from the “complexHeatmap” package (33) in R. Since there was no explicit argument in the Heatmap () to scale the rows/columns, we scaled our expression matrix using the scale () (28) and then constructed the heatmap of the resulting scaled matrix. The Volcano plot and the MA plot were generated using the volcanoplot () and plotMA () of the limma package (29), respectively.

### Variable Selection Methods

The processed microarray derivation dataset had 20,174 genes for 228 samples. Our next objective was to reduce the dimensionality of the dataset by removing redundant variables. To do that, we used three commonly used variable selection techniques, including random forest-based variable importance, LASSO, and Minimum Redundancy and Maximum Relevance. The variables generated by each of the above three methods were pooled together into one aggregated variable set, and the list of 17 differentially expressed genes were also added to that list. The Pediatric Risk of Mortality Score (PRISM) for each patient was computed and included in the model (34). For external validation, we used both LASSO based and tree-based variable selection techniques (using the extra trees classifier with 250 estimators) to select the top variables for further analysis.

### Classification Models

Imbalanced data can negatively affect learning algorithms. Hence, in this study, we experimented with both oversampling and undersampling techniques to balance the training data. Among the undersampling techniques, we implemented Cluster centroids (CCN), Repeated Edited Nearest Neighbors (REDN), Edited Nearest Neighbors (EDN), Instance Hardness Threshold (IHT), and Random Undersampling (RUS). Cluster centroids (35) undersamples the majority class by first calculating the centroid of the majority class and then finding all the instances nearest to this centroid in the input variable space. Consequently, instances far away from the centroid are discarded. The scikit-learn implementation of this method uses the KMeans algorithm to find the cluster centroids. In case of the Edited Nearest Neighbor resampling technique (36), all instances whose class label differs from that of half of its k-nearest neighbors are discarded. In Repeated Edited Nearest Neighbors, the EDN technique is successively applied until no further instances can be removed from the majority class. The Instance Hardness Threshold undersampling technique (37) works by successively applying a set of *n* learning algorithms on the training set followed by removing those instances that are frequently misclassified. Random Undersampling involves randomly selecting instances from the majority class to remove from the training set. SMOTE (Synthetic Minority Oversampling Technique) (38) is a frequently used oversampling technique that works by synthesizing new instances of the minority class from existing data. Specifically, a data point (say *a*) from the minority class is first chosen at random and its k-nearest neighbors are identified. A randomly selected neighbor (say *b*) is then chosen and a synthetic example is created at a randomly selected point between *a* and *b*.

We then developed machine learning algorithms using several tree-based classifiers and logistic regression. The two tree-based classifiers were the Balanced Random Forest (BRF) and Extra Trees (ET) classifier. A random forest classifier combines the predictions from hundreds of decision trees built on random bootstrapped samples of the dataset using a random subset of variables when splitting the nodes. In case of imbalanced classification, a balanced random forest classifier balances data by randomly undersampling each bootstrapped sample. The extra trees classifier is a meta estimator that fits a user-defined number of randomized trees (base models) on different sub-samples of the data and then combines the predictive power of these models into one optimal model. In case of binary classification, a Logistic Regression (LOGIT) classifier works by calculating a linear combination of the log-transformed gene expression values across samples and generating a linear decision boundary to separate the two classes from one another. For the external validation analysis, in addition to the above classifiers, we also used three ensemble techniques, namely the Gradient Boosting classifier (GB), Easy Ensemble (EE), and XGBoost classifier. All three classifiers work by combining several base learning models into a strong predictive model. Gradient Boosting (39) aims to reduce the loss of the model by iteratively adding weak learners in a stagewise fashion using a gradient descent approach and XGBoost (Extreme Gradient Boosting) (40) is a fast and highly efficient implementation of the Gradient Boosting method that uses L1 and L2 regularization to generate more generalizable models. The Easy Ensemble classifier (41) is essentially a collection of AdaBoost learners trained on different random bootstrap samples of the training set. A general framework explaining how tree-based classifiers and logistic regression models work is available in the Supplementary Document.

### Model Selection and Tuning

**Figure 1:**
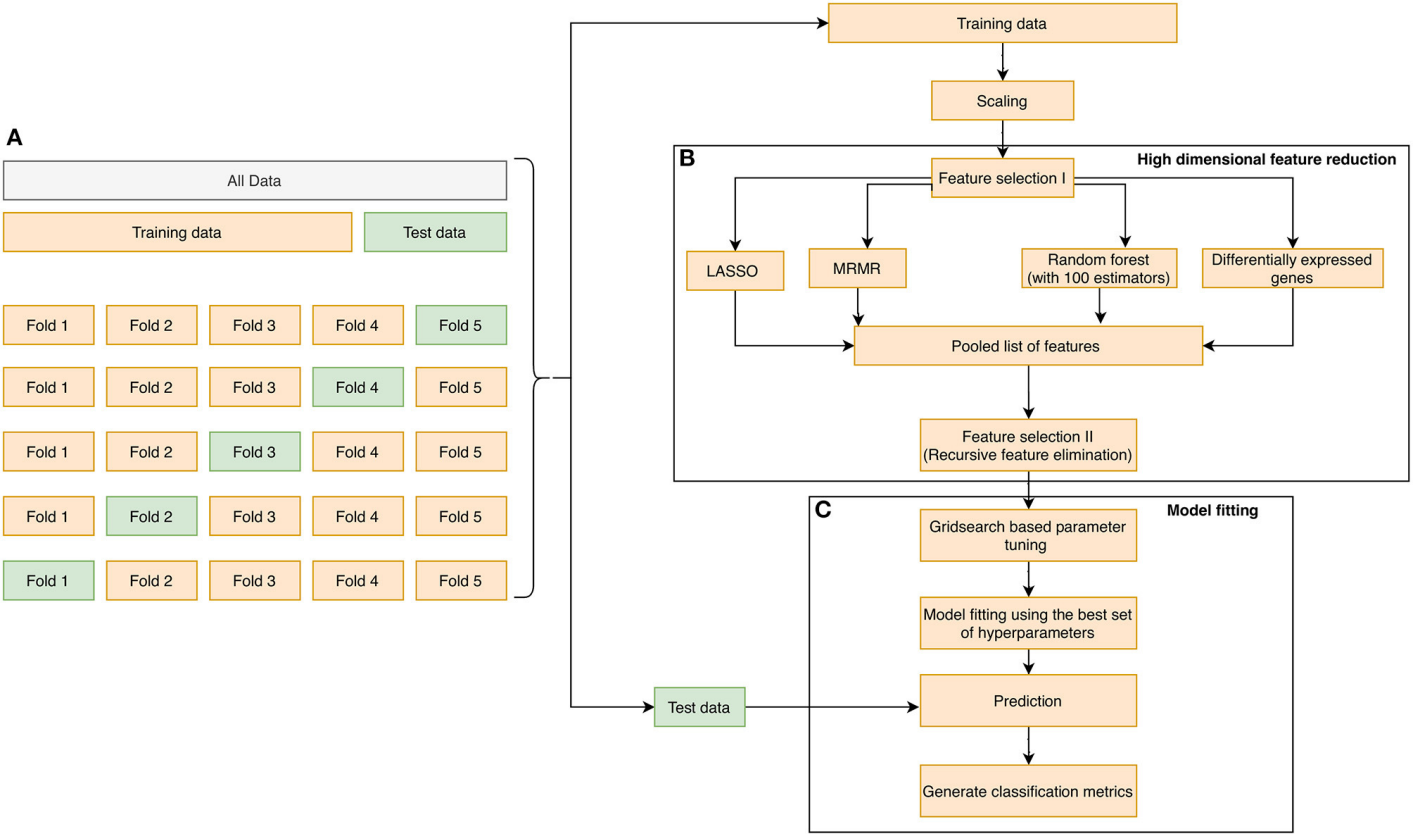
The overall methodology design for biomarker discovery from the derivation dataset GSE66099 containing 228 samples is illustrated in this figure. **(A)** The initial data is aggregated, normalized, corrected for batch normalization, and separated into even chunks using k-fold cross-validation (CV). In our pipeline, we used *k* = 5. **(B)** The training chunks of the CV are used for model development; the data analysis pipeline follows the Complete Cross-Validation (CCC) approach defined by Alder et al. (42). In addition to DEG, we apply three other variable selection methods to generate a pool of candidate genes. We then apply a wrapper method, namely the RFE to arrive on the most predictive genes. **(C)** The genes selected by the RFE method are then used to develop a predictive model. The model is then evaluated on the test fold of the CV. This process is repeated for the remaining training and test folds. Finally, the entire 5-fold CV is repeated 10 times to generate a total of 50 iterations, and the top predictors from **(B)** are saved and analyzed to generate a normalization score, which is a measure of how often a gene appears as a top predictor across each of the 50 iterations.

The learning process that was adopted in the manuscript is illustrated in Figures 1A–C. First, the dataset was randomly partitioned in a stratified fashion into five equal subsets (Figure 1A). Four of the five subsets were combined into one training set. In each training phase, we performed (1) variable selection (Figure 1B): The pooled list of variables using the three variable reduction approaches (described in the previous section), along with statistical filtering by DEG, were obtained. Finally, Recursive Feature Elimination (RFE) (43) was performed on the pooled variables to remove redundancies and arrive at a subset of the most important genes. (2) Hyperparameter tuning was performed using a cross-validated grid search technique over a parameter grid using the AUROC metric as the scoring function. (3) A classifier was trained using the hyperparameters, and the corresponding prediction scores were obtained on the hold-out test set (Figure 1C). We repeated the entire process 10 times, resulting in 50 unique train and test splits. The classification performances obtained during each run were averaged, and the mean scores were reported. We assessed the performance of our classifiers using four widely used performance metrics: Sensitivity, Specificity, Mathews correlation coefficient, and Area under the ROC curve. They were defined as follows:

*Condition Positive (P):* The number of complicated course patients in the dataset
*Condition Negative (N):* The number of uncomplicated course patients in the dataset
*True Positives (TP):* The generated model has *correctly* predicted the positive class or the complicated course patients.
*True Negatives (TN):* The generated model has *correctly* predicted the negative class or the uncomplicated course patients.
*False Positives (FP):* The generated model has *incorrectly* predicted the positive class or the complicated course patients.
*False Negatives (FN):* The generated model has *incorrectly* predicted the negative class or the uncomplicated course patients.
*Sensitivity (or True Positive Rate):* A quantity that measures the proportion of the complicated course patients that are correctly identified. Mathematically, this can be expressed as:
  $$\begin{array}{l} Sensitivity=\frac{TP}{TP+FN} \end{array}$$
*Specificity:* A quantity that measures the proportion of the uncomplicated course patients that are correctly identified. Mathematically, this can be expressed as:
  $$\begin{array}{l} Specificity=\frac{TN}{TN+FP} \end{array}$$
*False Positive Rate:* A quantity that is defined as the ratio between the number of uncomplicated patients incorrectly categorized as complicated (false positives) and the actual number of uncomplicated patients (before classification).
  $$\begin{array}{l} False\;Positive\;Rate=\frac{FP}{FP+TN} \end{array}$$
*Mathews correlation coefficient:* A quantity that measures the quality of binary classification models. Mathematically, this can be expressed as:
  $$\begin{array}{l} MCC=\frac{TP\times TN-FP\times FN}{\sqrt{\left(TP+FP\right)\left(TP+FN\right)\left(TN+FP\right)\left(TN+FN\right)}} \end{array}$$
  The value of MCC ranges from −1 to 1 where −1 signifies a perfect misclassification and vice versa. Accuracy and F1 score are widely used performance metrics that can show overoptimistic inflated results for imbalanced data and hence were not included in our study.
*Area under the ROC curve:* A probabilistic binary classifier outputs the probability of a particular data point belonging to a class. To map these probabilities to a binary category, a classification threshold is usually defined. A value above that threshold will be classified as “positive” and vice versa. An ROC curve plots True Positive Rate vs. False Positive Rate and displays the performance of a binary classifier at different classification thresholds. Area under the ROC curve (or AUROC) measures the entire two-dimensional area under the ROC curve. Basically, it gives an aggregate measure of the performance of a binary classifier at various classification thresholds. Another way of interpreting AUROC is as the probability that a classifier ranks a random positive instance more highly than a random negative instance. AUROC ranges from 0 to 1 where 0 signifies a perfectly inaccurate classifier and vice versa. The ROC plots displaying the performance of the binary classifiers were generated using the matplotlib (44) plotting library in Python. A detailed discussion on each step of the workflow outlined in Figure 1 is available in the Supplementary Document.

To study the predictive power of our top 20 gene variables from the stability analysis, we performed validation on independent test sets. In the first step, we applied different undersampling and oversampling techniques on the derivation dataset. Then we performed a LASSO based variable selection to remove redundant variables. The hyperparameters of the classifier were tuned using the 5-fold cross-validation grid search technique over a parameter grid using the AUROC metric as the scoring function. The tuned classifier was then trained on the derivation set, and the corresponding prediction scores were obtained on the hold-out validation sets (GSE54514, E-MEXP-3850, E-MEXP-3567, and GSE40586). The predicted probabilities from a classification model are converted to discrete class labels using a parameter called “classification threshold.” Consequently, if “*x*” was the reported classification threshold for a given classifier, then all instances with predicted probabilities greater than “*x*” were classified as having a complicated course outcome and vice versa. To further fine-tune the classifiers, instead of using the default classification threshold of 0.5, we experimented with different thresholds between 0 and 1 with step sizes of 0.001. This is particularly important for an imbalanced classification problem like ours and must be taken into account to make sure that the minority class examples are predicted correctly.

The same set of performance metrics were used to assess the validation classifier's quality, as stated in the previous section.

### Distribution of Variables

To study the class-wise distributional differences between the variables (or genes), we conducted the two-sample Kolmogorov-Smirnov (KS) test using scipy stats module. The distplot function from the seaborn package (45) was used to visualize the class-wise Gaussian kernel density estimate of each of the variables. In case of the E-MEXP-3567 dataset, owing to the small sample size (only 12 samples), we adopted a repeated resampling with replacement (or bootstrapping) technique to estimate the KS statistic values. The formulation of the KS test and an extended discussion on KDE plots is available in the Supplementary Document.

## Results

### Clinical Characteristics of the Patients Included in the Derivation Dataset

Out of the 229 pediatric septic patients present in the derivation dataset (GSE66099), 52 had a complicated course outcome. One patient was excluded due to missing the outcome variable. The age of the cohort was 3.81 ± 3.42 (mean ± SD) years. Out of the 228 patients in the cohort, 18 patients met the criteria for sepsis, 30 for Systemic Inflammatory Response Syndrome (SIRS), and 180 for septic shock. Males constitute the majority of the dataset (139; 61%; *P* = 0.00093). In the 52 complicated course cohort, 31 (59.6%; *P* = 0.16) were male; whereas in the non-complicated course group, 108 (61.36 %; *P* = 0.0026) were male. Of the 52 with complicated courses, 28 (53.8%; *P* = 0.58) died. The clinical characteristics of all patients with a complicated or uncomplicated course outcome are provided in Supplementary Table 1A. Microbiologic results are shown in Supplementary Table 3. The most frequently identified organisms were *Staphylococcus aureus* (in 22 patients; 9.65%), *Pneumococcus* (in 18 patients; 7.9%), *Group A Strep* (in 14 patients; 6.14%), and *Klebsiella* (in 11 patients; 4.82%).

### Normalization and Subsequent Batch Correction Removed Unwanted Variations From the Derivation Dataset

**Figure 2:**
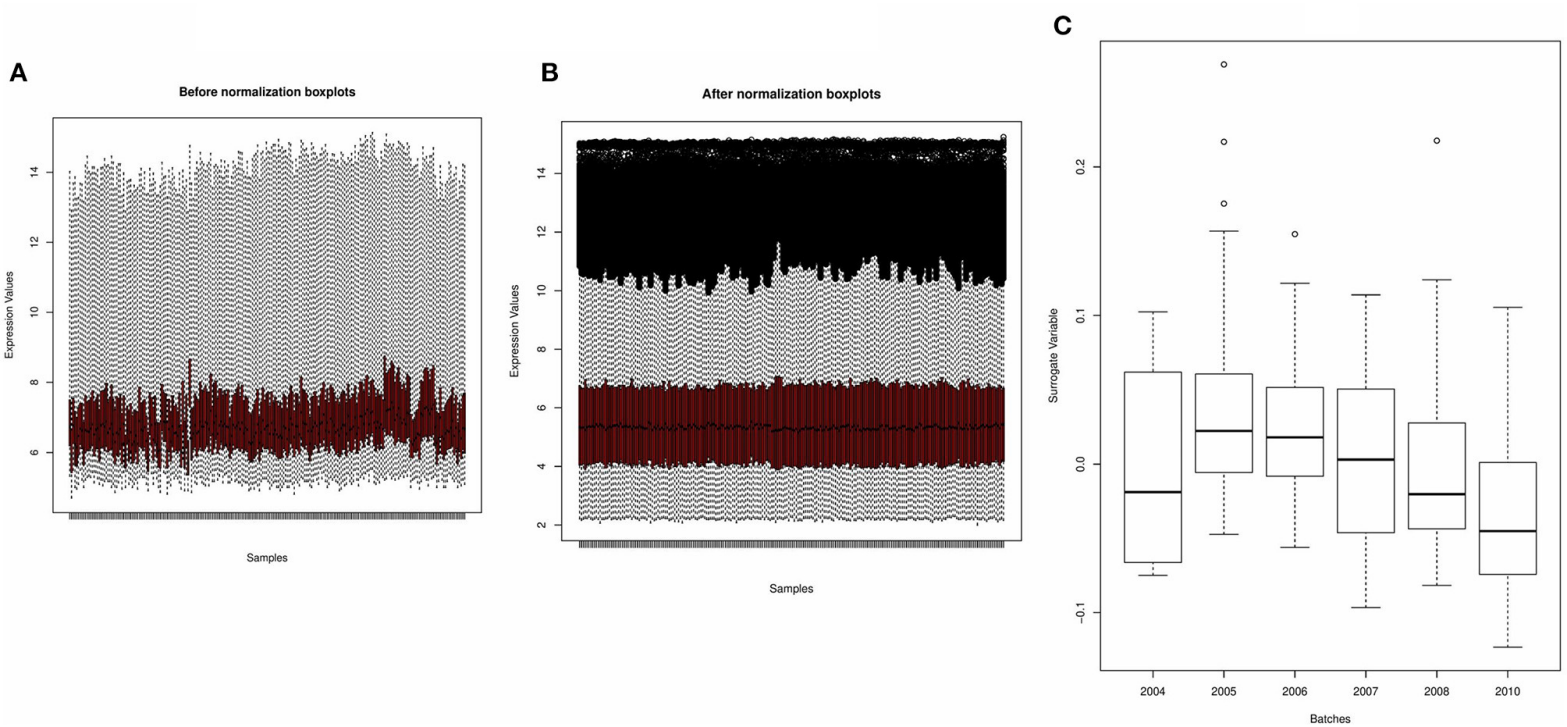
Preprocessing of derivation dataset GSE66099 containing 228 samples. **(A,B)** Average gene expression values before normalization and after normalization. The x-axis represents the samples, and the y-axis represents the gene expression values. According to the figures, the average expression values of the samples were more stable and consistent after normalization and suitable for analysis. **(C)** One of the most well-known sources of variation in gene expression studies is batch effects when samples are processed during different time points or by different groups of people. We removed the batch effects from the data due to the microarray experiments being conducted over multiple years using the Combat() in the “sva” package. In the given figure, the first SVA component ordered by date before batch effect correction shows that one of the inferred batch effects (or the surrogate variable) is associated with the actual batch variable.

Figures 2A,B shows the boxplots of the average gene expression of samples derived from the derivation dataset GSE66099 before and after normalization. Before normalization, the expression values had inconsistent distributions. After normalization all the samples were aligned at the overall mean and variance. The output of the SVA consisted of two surrogate variables and only one of them had a significant association with the actual batch variable. The boxplot drawn in Figure 2C shows that there is a relationship between the inferred batch effect and the observed batch effect. This is similar to performing an ANOVA test and checking if the values of the SVs calculated for each sample is different between the batches or whether there is a difference in the means between the boxplots. An expanded discussion on the normalization process and identification of batch effects is available in the Supplementary Document.

### Expression Levels of the DEGs Generated Prominent Clusters Separating the Complicated and Uncomplicated Course Patients

**Figure 3:**
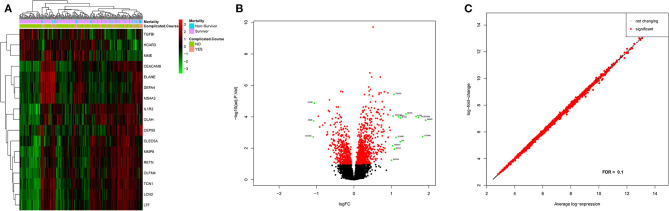
Differential gene expression analysis results of the patients included in the derivation dataset GSE66099 containing 228 samples. **(A)** Heatmap representing differentially expressed genes between complicated and uncomplicated course groups with annotations. The color and intensity of the boxes represent changes in gene expression. Red represents upregulated genes and green represents downregulated genes. The horizontal bars show annotations for complicated course outcomes and mortality and are useful for interpreting the sample-wise clusters formed using the expression measurements. **(B)** Volcano plot of differentially expressed genes in complicated and uncomplicated course outcomes. A volcano plot helps us to assess the adjusted *p*-values (significance), and the log fold changes (biological difference) of differential expression for the given list of genes at the same time. **(C)** An MA plot is a 2D scatter plot (each dot representing a gene) that represents log fold change vs. mean expression across two different conditions. All the significantly differentially expressed genes (FDR cutoff = 0.1) are colored in red and the genes without significant gene expression differences are colored in black.

Based on an adjusted *p*-value cutoff <0.1, a total of 1,269 differentially expressed genes (DEGs) from 20,174 were found between the complicated and uncomplicated group of patients, including 808 upregulated genes and 461 downregulated genes. The DEGs with an absolute log2 fold change of at least one (*n* = 17) are shown in Supplementary Table 2. For the complete list, refer to Supplementary Table 4. Figure 3A illustrates the heatmap of 17 DEGs (absolute log2 fold change >1 and adjusted *p* < 0.1) across the complicated *vs*. uncomplicated groups derived using the derivation dataset GSE66099. The samples are represented on the vertical axis and the genes are on the horizontal axis. The normalized gene expression values of the DEGs were used to construct the heatmap. Red and green represent the upregulated and downregulated genes, respectively. Both the samples and genes were clustered to give a better idea of the groups formed using the expression values. Two horizontal bars show annotations for the complicated course outcomes and mortality.

Three prominent sample clusters were observed from the heatmap. The first cluster contained none of the 52 complicated course patients, while the second cluster contained nine (17%) of the 52 complicated course patients and the third contained 43 (83%) of the complicated course patients. A similar cluster trend is noticed among survivors/non-survivors. None of the non-survivors were in the first cluster, four (14%) out of 28 non-survivors were in the second cluster and the remaining 24 (86%) were in the third cluster.

Genes were also clustered into two distinct groups of upregulated and downregulated genes. *MME, TGFBI*, and *HCAR3* together formed one cluster of downregulated genes while the rest were in the upregulated category. *LTF* and *IL1R2* were the most highly expressed genes, whereas *CEP55*, and *MS4A3* were the least expressed genes. *MMP8* was the most upregulated gene while *HCAR3* was the most downregulated gene.

Figure 3B shows the volcano plot of significantly different genes. Only genes having absolute log2 fold change >1 and adjusted *p* < 0.1 were considered differentially expressed. Excluding the above-mentioned downregulated genes, green dots represent log2 fold change >1 and adjusted *p* < 0.1; red dot represents genes with adjusted *p* < 0.1 but log2 fold change between −1 and 1; whereas black represent genes with log2 fold change between −1 and 1 and adjusted *p* > 0.1. Also, in Figure 3C, an MA-plot displays the log2 fold change between complicated course and non-complicated course samples as a function of the average expression level across all samples. Red dots are relatively larger than the black ones. The dataset included a total of 30 uncomplicated disease course patients who met the SIRS criteria when excluding these patients from our analysis we observed five genes that achieved robust normalization scores but did not meet the DEG cutoffs. Those genes were: *TGFBI, DEFA4, CEP55, MME*, and *OLAH*.

### Functional Enrichment Analysis of the DEGs Displayed an Association With an Overactive Innate Immune System and Neutrophil Activity

**Figure 4:**
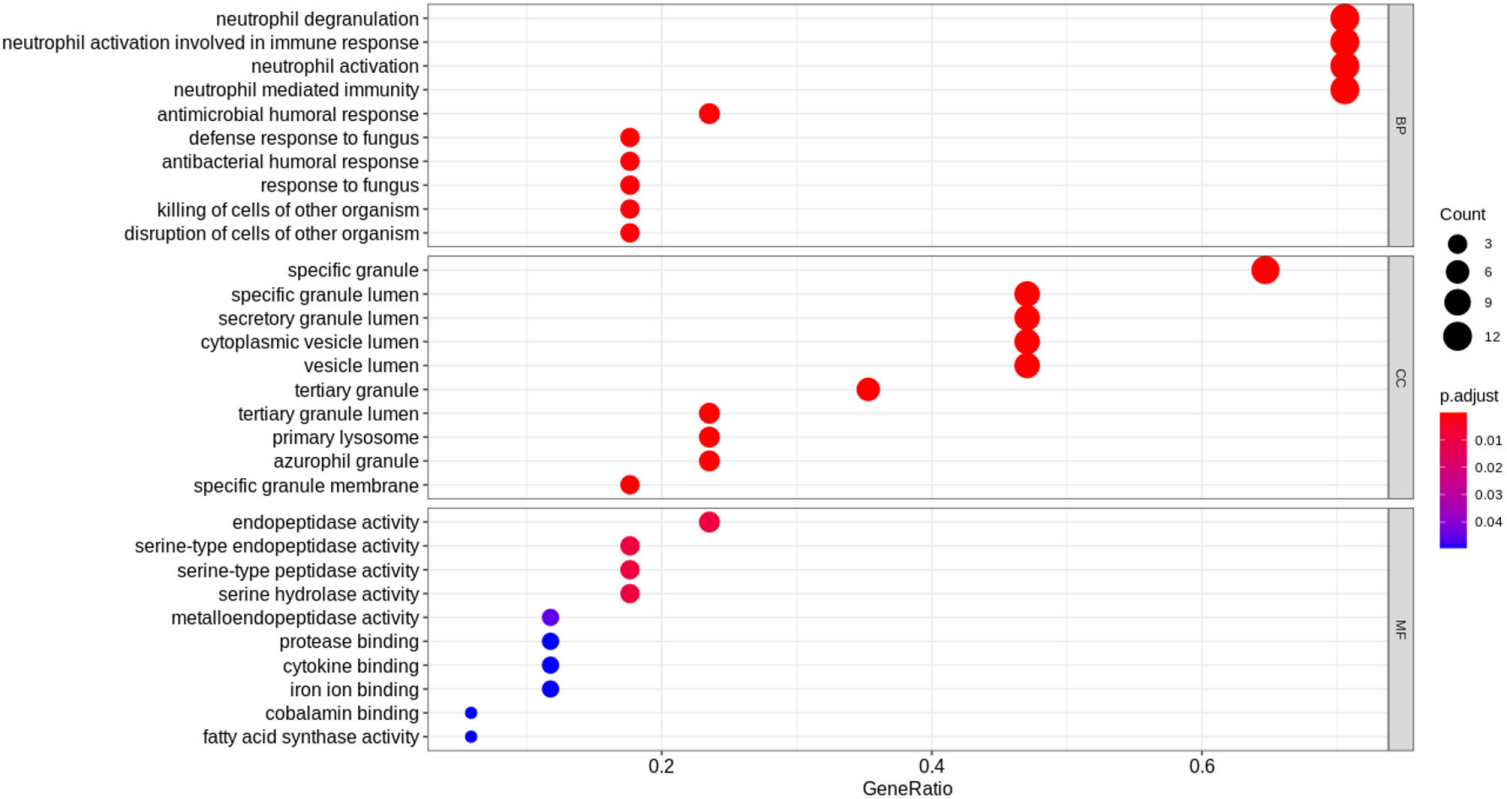
Functional analysis of the 17 DEGs using the expression levels of the patients included in the derivation dataset GSE66099 containing 228 samples. The Gene Ontology terms representing our knowledge for the biological domain are grouped on the basis of three major aspects: Biological Processes (BP), Cellular Components (CC), and Molecular Function (MF). A biological process is a specific objective that an organism is genetically designed to accomplish. It is often described by the ending state or the outcome. For instance, a biological process defined as “cell division” results in the creation of a divided cell (two daughter cells) from a single parent cell. A Cellular Component (CC) defines a location occupied by a macromolecular machine during the execution of a specific molecular function. For instance, “cytoplasmic side of plasma membrane” is a cellular component defining the location of a gene product relative to cellular structures. A Molecular Function (MF) represents the primary activity of a gene product at the molecular level. Biochemical activities such as “binding” or “catalysis” are examples of GO terms representing molecular functions. In this figure, the y-axis represents the gene ontology terms and the x-axis represents the “Gene Ratio” or the percentage of total DEGs in the given GO term. The size of the dot or the “Gene Count” represents the number of genes associated with the enriched term and the color of the dot represents the significance of the terms (more significant terms being redder). “P.adjust” is the *p*-value adjusted using the Benjamini-Hochberg procedure.

The top 17 most significantly differentially expressed genes (absolute log fold change >1 and adjusted *p* < 0.1) obtained from the derivation dataset GSE66099 were analyzed. “Hematopoietic cell lineage” (hsa04640) was the most enriched KEGG pathway; “neutrophil degranulation” (HSA-6798695), “immune system” (HSA-168256) and “antimicrobial peptides” (HSA-6803157) were some of the most enriched Reactome pathways. Gene Ontology (GO) analysis of the DEGs revealed known GO terms such as “neutrophil degranulation” (GO:0043312), “neutrophil activation involved in immune response” (GO:0002283), “neutrophil activation” (GO:0042119), and “neutrophil-mediated immunity” (GO: 0002446) as some of the top enriched biological processes; “Serine-type endopeptidase activity” (GO:0004252), “endopeptidase activity” (GO: 0004175), and “serine-type peptidase activity” (GO:0008236) were some of the most enriched molecular functions; “Specific granule” (GO:0042581) and “specific granule lumen” (GO:0035580) were some of the most enriched cellular components. Figure 4 displays the results from the functional analysis. The y-axis represents the gene ontology terms and the x-axis represents “Gene Ratio” or the percentage of total DEGs in the given GO term.

We also performed functional analysis on the top predictors from our machine learning analysis which included three additional genes (*PLCB1, NLRP1*, and *SDC4*) apart from the DEGs (Table 1). The top enriched biological process term was neutrophil degranulation (GO:0043312) similar to the results from our previous functional analysis with the DEGs. Some of the newer enriched terms included cell activation (GO:0001775), immune response (GO: 0006955), immune system process (GO:0002376), and response to stimulus (GO: 0050896). It is well-known that immunosuppression is a hallmark of sepsis and the top predictors capable of distinguishing between complicated and uncomplicated course patients show enrichment in the immune response process. Neutrophil degranulation (HSA-6798695), innate immune system (HSA-168249) were among the top enriched REACTOME pathways. The innate immune system is activated as the first response to an infection before the adaptive immune system. Most of the clinical phenotypes of sepsis can be attributed to the innate immune response. Interestingly, the adaptive immune response was not one of the enriched pathways for our list of genes which might warrant further investigation.

**Table 1:** Top consistently chosen variables across folds.

**Variable name**	**Normalized score (%)**	**Full gene name**	**Overlap with known gene sets**	**Adj *p*-value (from DEG analysis)**	**KS test**	
					**Statistic**	***p*-value**
*RETN*	100	Resistin	PERSEVERE (14)	6.65 × 10^−5^	0.44	2.08 × 10^−7^
*IL1R2*	100	Interleukin 1 Receptor Type 2	Sweeney et al. (17)	1 × 10^−4^	0.34	1 × 10^−4^
PRISM Score^**^	100	Not applicable	Not applicable	Not applicable	0.46	3.08 × 10^−8^
SDC4^*^^#^	100	Syndecan-4	Nikaido et al. (48)	1.93 × 10^−10^	0.49	3.98 × 10^−9^
*TGFBI*	100	Transforming Growth Factor Beta Induced	PERSEVERE XP (15), Sweeney et al. (17)	1.36 × 10^−5^	0.41	1.15 × 10^−6^
*CEACAM8*	96	CEA Cell Adhesion Molecule 8	Sweeney et al. (17)	1 × 10^−4^	0.44	1.35 × 10^−7^
*MMP8*	92	Matrix metalloproteinase-8	PERSEVERE (14)	2 × 10^−4^	0.39	4.51 × 10^−6^
*ELANE (ELA2)*	92	Elastase, Neutrophil Expressed	PERSEVERE (14)	2 × 10^−3^	0.37	1.59 × 10^−5^
CLEC5A^*^	92	C-Type Lectin Domain Containing 5A		8.87 × 10^−5^	0.37	2.35 × 10^−5^
TCN1^*^	92	Transcobalamin 1		1 × 10^−4^	0.39	6.59 × 10^−6^
*CEP55*	92	Centrosomal Protein 55	PERSEVERE XP (15), Sweeney et al. (17)	3.78 × 10^−6^	0.40	2.20 × 10^−6^
*MME*	92	Membrane Metalloendopeptidase	PERSEVERE XP (15)	2 × 10^−4^	0.39	4 × 10^−6^
MS4A3^*^	92	Membrane Spanning 4-Domains A3		7 × 10^−3^	0.34	1 × 10^−4^
*DEFA4*	92	Defensin Alpha 4	Sweeney et al. (17)	5.8 × 10^−2^	0.30	1 × 10^−3^
HCAR3^*^	88	Hydroxycarboxylic Acid Receptor 3	Kangelaris et al. (49)	2 × 10^−3^	0.33	1 × 10^−4^
*LCN2*	88	Lipocalin-2	PERSEVERE (14)	9.6 × 10^−5^	0.39	3.8 × 10^−6^
OLAH^*^	84	Oleoyl-ACP Hydrolase Olfactomedin 4	Basu et al. (50)	1.1 × 10^−2^	0.31	6 × 10^−4^
*OLFM4*	68	Olfactomedin 4	Basu et al. (50)	1.8 × 10^−3^	0.33	1 × 10^−4^
*LTF*	68	Lactoferrin	PERSEVERE (14)	3.9 × 10^−3^	0.34	9.5 × 10^−5^
PLCB1^*^^#^	68	Phospholipase C Beta 1			0.3	1 × 10^−4^
NLRP1^*^^#^	68	NLR Family Pyrin Domain Containing 1		2.56 × 10^−6^	0.45	5.41 × 10^−8^

* *Novel genes*.

# *Non-DEGs*.

** *Clinical variable. Novel Biomarkers are indicated in bold*.

### Machine Learning Models Built Using the Derivation Dataset Generate a Strong Predictive Model of Complicated Sepsis Based on the Top Gene and Clinical Variables

**Figure 5:**
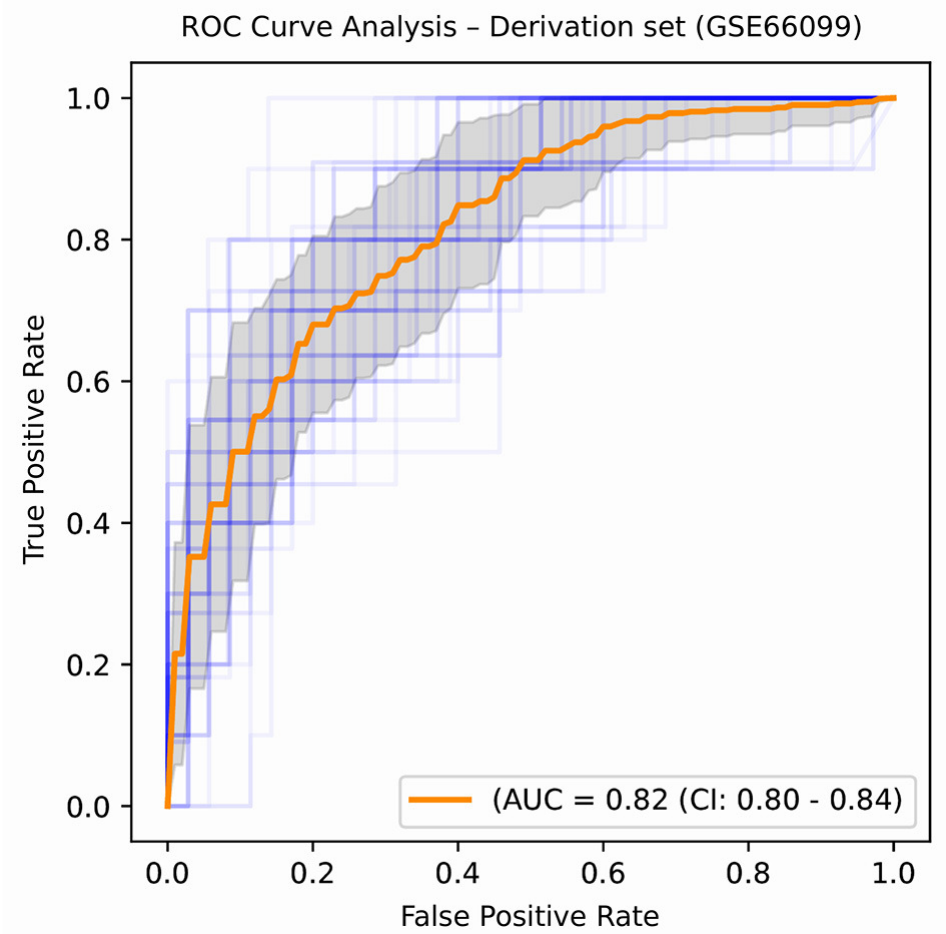
ROC plot displaying the classification performances of the best model (in terms of mean AUROC) trained using the top 21 consistently chosen gene and clinical variables (Table 2) from patients included in the derivation dataset GSE66099 containing 228 samples. An ROC plot illustrates the performance of a binary classifier at different classification thresholds usually featuring a false positive rate (1-Specificity) on the x-axis and true positive rate (Sensitivity) on the y-axis. The top left corner is an ideal point with a false positive rate of zero and a true positive rate of one. The area under the curve (or AUC) denotes the probability that a randomly chosen positive instance is ranked higher than a randomly chosen negative one by our classifier. An AUC of zero means that the classifier is predicting the positive class as negative and vice versa, while an AUC of one denotes perfect separability. In the above figure, we denote the ROC plots generated from the different cross-validation experiments along with the mean area under the curve (in orange). The variance of the curve (shaded part) roughly shows how the output from our best performing model is affected by changes in the training data.

An ROC curve plots True Positive Rate vs. False Positive Rate and displays the performance of a binary classifier at different classification thresholds. Area under the ROC curve (or AUROC) measures the entire two-dimensional area under the ROC curve and estimates the ability of a binary classifier at discriminating between the two classes (complicated and uncomplicated course outcome). AUROC ranges from 0 to 1 and higher values of AUROC signifies better discriminative power of the generated classifier. The best performance in terms of overall mean AUROC (=0.823), mean specificity (=0.885) and mean MCC (=0.445) was obtained using an oversampling technique, SMOTE, and BRF classifier pair. The ROC curve of the best performing model is shown in Figure 5 and the results for the best performing classifiers is shown in Table 3. The orange-colored curve is the mean ROC curve obtained after averaging the results from different runs of the repeated 5-fold cross-validation. The list of the top clinical and genomic variables that were consistently chosen across different folds of the cross-validation of the top-performing model and their overlap with previously published predictor genes in sepsis is shown in Table 1. PRISM score, which was the only clinical variable included in our model was also chosen as one of the top predictors in our analysis. The “normalized score” represents the fraction of times the given predictor was consistently chosen over all experiments, representing the stability (46) of these genes as possible predictors of a complicated course. On the derivation cohort GSE66099 containing 228 patients, the best model in terms of mean AUROC (=0.823) was obtained using the top consistently chosen 20 gene biomarkers and PRISM score. Seventeen out of these 20 gene biomarkers were among the differentially expressed genes as shown in the heatmap and the volcano plot (green and orange dots) in Figure 3 and Table 1. Among the other classification metrics, the best mean sensitivity (=0.792) was obtained using an undersampling technique, REDN, and the BRF classifier (Table 3).

**Table 2:** Top genomic predictors obtained during external validation analysis.

**Validation dataset identifier**	**Cohort type**	**Best AUROC**	**Gene names**
GSE54514^#^	Adult	0.723	*MMP8, CEACAM8, LCN2, RETN, CLEC5A, TGFBI, CEP55, MME, OLAH, SDC4*
E-MEXP-3850^*^	Pediatric	0.956	*MMP8, TCN1, OLAH, CEP55, PLCB1, OLFM4, HCAR3, TGFBI, MS4A3, CEACAM8, SDC4*
E-MEXP-3567^#^	Pediatric	0.83	*MMP8, CEACAM8, LCN2, RETN, CLEC5A, TGFBI, CEP55, MME, OLAH, SDC4*
GSE40586^#^	MIxed	0.822	*MMP8, OLFM4, CEACAM8, RETN, LTF, HCAR3, IL1R2, MS4A3, TGFBI, DEFA4, MME, OLAH, SDC4, PLCB1, NLRP1*

* *Kolmogorov-Smirnov test was performed on the ranked list of genes obtained using the tree-based variable importance method. Only the significant (p < 0.1) results are included in this list*.

# *List of genes selected using the LASSO-based variable selection technique*.

**Table 3:** Performance measure for various sampling technique and classifier pairs (Derivation set).

**Sampling technique—classifier**	**Sensitivity (CI)**	**Specificity (CI)**	**AUROC (CI)**	**MCC (CI)**
CCN-BRF	0.722 (0.700–0.743)	0.740 (0.722–0.755)	0.802 (0.787–0.823)	0.411 (0.383–0.434)
CCN-ET	0.700 (0.689–0.711)	0.752 (0.733–0.767)	0.801 (0.767–0.834)	0.404 (0.378–0.423)
CCN-LOGIT	0.624 (0.578–0.639)	0.822 (0.810–0.834)	0.818 (0.800–0.835)	0.424 (0.381–0.450)
REDN-BRF	**0.792 (0.776–0.811)**	0.647 (0.631–0.657)	0.801 (0.765–0.833)	0.374 (0.361–0.394)
REDN-ET	0.707 (0.678–0.723)	0.722 (0.710–0.741)	0.815 (0.799–0.834)	0.376 (0.351–0.389)
REDN-LOGIT	0.780 (0.765–0.799)	0.691 (0.667–0.712)	0.820 (0.801–0.839)	0.409 (0.387–0.420)
SMOTE-BRF	0.551 (0.497–0.584)	**0.885 (0.878–0.903)**	**0.823 (0.800–0.838)**	**0.445 (0.411–0.476)**
SMOTE-ET	0.554 (0.532–0.578)	0.865 (0.842–0.878)	0.822 (0.800–0.837)	0.422 (0.380–0.449)
SMOTE-LOGIT	0.527 (0.499–0.537)	0.859 (0.839–0.870)	0.790 (0.765–0.801)	0.399 (0.367–0.411)

*Top average values obtained for each classification metric are indicated in bold*.

### Clinical Characteristics of the Patients Included the Validation Dataset

For our first validation dataset (GSE54514), out of the 127 septic patients, 27 had a complicated course outcome. The age of the cohort was 59.13 ± 15.99 (mean ± SD). Females constitute the majority of the dataset (75; 59%; *P* = 0.11). In the 27 complicated course patients, 14 (51.85%; *P* = 0.02) were females; whereas, in the non-complicated course group, 61 (61%; *P* = 0.14) were females. Of the 27 with the complicated course, 17 (63%; *P* = 0.16) died. For our second validation dataset (E-MEXP-3850), gene expression data from five critically ill children was measured at 29 distinct time points during the first 48 h of admission to the PICU. Of the 5 patients, only one patient died within 28 days of admission to the hospital. The age of the cohort was 1.3 ± 0.58 (mean ± SD). The majority of the patients were females (3; 60%; *P* = 0.6). For the third validation dataset (E-MEXP-3567), the average age of the cohort was 2.0 (IQR 0.6–6.9). There was an equal number (six) of male and female infants in the cohort. The majority of the deceased patients were females (4; 67%; *P* = 0.33). For the fourth validation dataset (GSE40586), out of the 21 patients suffering from bacterial meningitis, 8 had a complicated course outcome. The age of the cohort was 43.4 ± 26.86. There was no information regarding the gender distribution of the cohort. Of the 8 patients with the complicated course, 2 (25%; *P* = 0.01) died. The clinical characteristics of patients belonging to the different validation cohorts are provided in Supplementary Tables 1B–E.

### Machine Learning Models Generated Using the Top 20 Gene Biomarkers Displayed High Classification Performances on Four Independent Test Datasets

**Figure 6:**
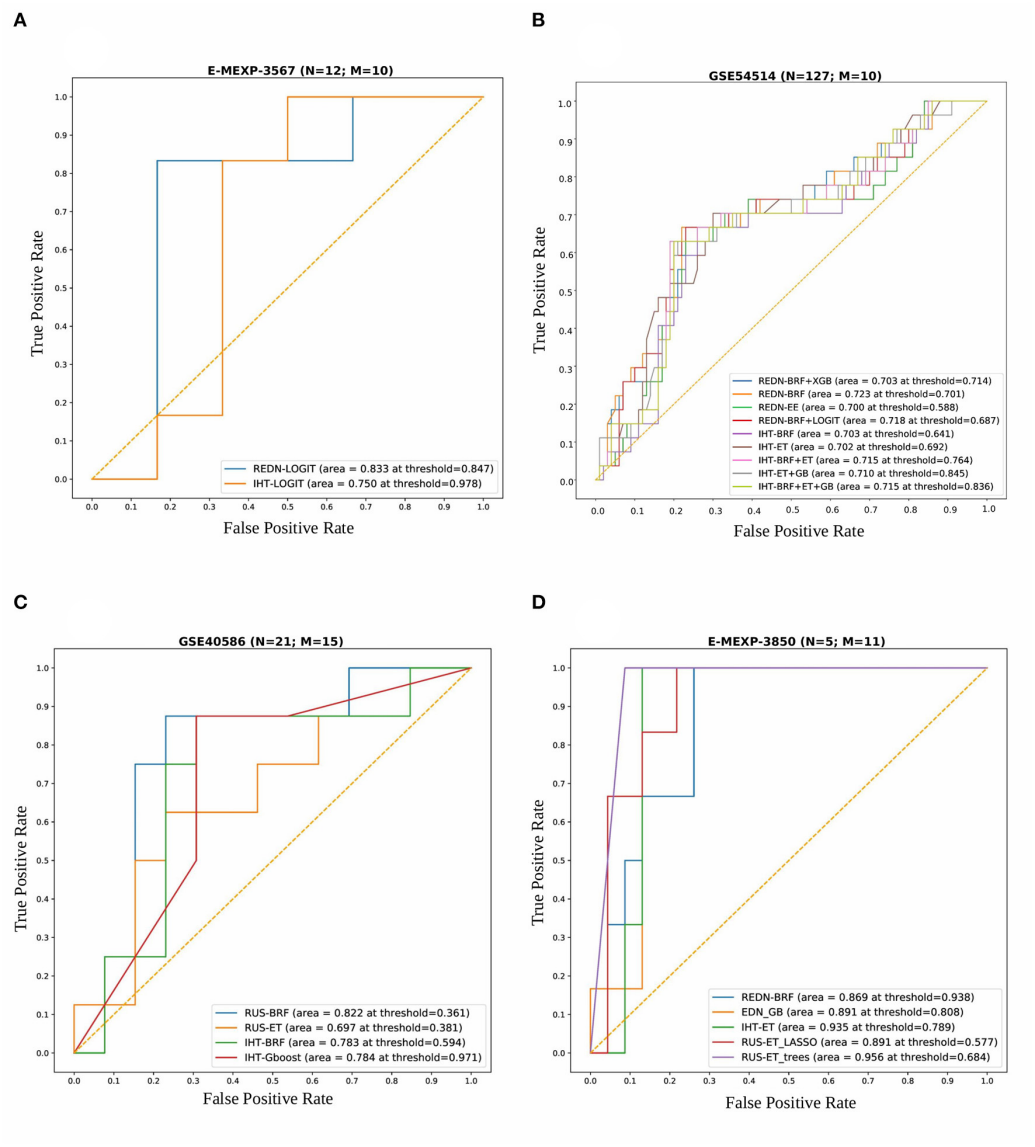
Combined ROC plots illustrating the performances of various binary classifiers on four independent validation sets. “N” represents the total number of samples in each dataset and “M” represents the total number of genes used to derive the best classification models (in terms of AUROC). **(A)** E-MEXP-3567: The best model (AUROC=0.833) was obtained using a total of 10 out of the 20 gene biomarkers (*MMP8, CEACAM8, LCN2, RETN, CLEC5A, TGFBI, CEP55, MME, OLAH*, and *SDC4*). **(B)** GSE54514: The best model (AUROC = 0.723) was obtained using a total of 10 out of the 20 gene biomarkers (*MMP8, CEACAM8, LCN2, RETN, CLEC5A, TGFBI, CEP55, MME, OLAH*, and *SDC4*). **(C)** GSE40586: The best model (AUROC = 0.822) was obtained using a total of 15 out of the 20 gene biomarkers. **(D)** E-MEXP-3850: The best model (AUROC = 0.956) was obtained using a total of 11 out of the 20 gene biomarkers (*MMP8, TCN1, OLAH, CEP55, PLCB1, OLFM4, HCAR3, TGFBI, MS4A3, CEACAM8*, and *SDC4*). Due to the inherent imbalance in the training data (GSE66099) we tried different classification thresholds for the tuned classifiers and reported the ones that gave the best classification performances. The list of top performing classifiers included both individual and stacked classifiers. The legend displays the names of the sampling-classifier combinations, the AUROC and the classification thresholds that gave the top results in brackets.

For the Array Express dataset E-MEXP-3567 based on the pediatric cohort, the best performance in terms of overall AUROC (=0.83) was obtained using an undersampling technique, REDN, and LOGIT classifier pair. This model also gave the best specificity (=0.83) and MCC (=0.67). The best sensitivity (=1.0), however, was obtained using an undersampling technique REDN, and BRF classifier pair. The list of 10 genes that were chosen as top variables using the LASSO-based variable selection technique were *MMP8, CEACAM8, LCN2, RETN, CLEC5A, TGFBI, CEP55, MME, OLAH*, and *SDC4*. The ROC plots of the top performing models obtained using this dataset is shown in Figure 6A. The best classifier (AUROC = 0.83) was obtained using a classification threshold of 0.847.

For the GSE54514 expression data based on the adult cohort, the best performance in terms of overall AUROC (=0.7233) was obtained using an undersampling technique, REDN, and BRF classifier pair. This model also gave the best MCC (0.395). The best specificity (0.80) was obtained using another undersampling technique, IHT and a stacked classifier containing BRF, GB, and ET. The best sensitivity (0.70) was obtained using the sampling technique, IHT and ET classifier pair. Both these methods used a LASSO based variable selection strategy. For the undersampling technique REDN, the list of genes that were selected using the LASSO based variable selection technique were *MMP8, CEACAM8, LCN2, RETN, CLEC5A, TGFBI, CEP55, MME, OLAH*, and *SDC4* and for IHT the list of genes that were selected was *OLFM4, CEACAM8, LCN2, RETN, ELANE, HCAR3, IL1R2, CLEC5A, MS4A3, TGFBI, DEFA4, CEP55, MME, SDC4, PLCB1*, and *NLRP1*. The ROC plots of the top performing models obtained using this dataset is shown in Figure 6B. Predominantly two undersampling techniques, namely, Repeated Edited Nearest Neighbors and Instance Hardness Threshold gave consistently good performances in terms of AUROC. The best classifier (AUROC = 0.723) was obtained using a classification threshold of 0.701.

For the GSE40586 expression data based on a diverse group of patients (infants, children, and adults), the best performance in terms of overall AUROC (=0.822) was obtained using an undersampling technique RUS, and BRF classifier pair. This model also gave the best sensitivity (=0.875) and MCC (=0.626). The best specificity (=0.846), however, was obtained using the undersampling technique RUS and a stacked ensemble of BRF and ET classifiers. The list of top 15 genes selected by the LASSO technique were *MMP8, OLFM4, CEACAM8, RETN, LTF, HCAR3, IL1R2, MS4A3, TGFBI, DEFA4, MME, OLAH, SDC4, PLCB1*, and *NLRP1*. The ROC plots of the top performing models obtained using this dataset is shown in Figure 6C. The best classifier (AUROC = 0.822) was obtained using a classification threshold of 0.361.

For the Array Express dataset E-MEXP-3850 based on the pediatric cohort, the best performance in terms of overall AUROC (=0.9566) was obtained using an undersampling technique, RUS, and ET classifier pair. A tree-based variable ranking approach gave the best results in this case. The list of 11 genes selected by the tree-based technique were *MMP8, TCN1, OLAH, CEP55, PLCB1, OLFM4, HCAR3, TGFBI, MS4A3, CEACAM8*, and *SDC4*.

This model also gave the best specificity (=0.913), sensitivity (=1.0), and MCC (=0.828). The ROC plots of the top performing models obtained using this dataset is shown in Figure 6D. The best classifier (AUROC = 0.956) was obtained using a classification threshold of 0.684.

The classification metrics and the list of the top genomic predictors for the best performing models generated during the validation analysis are shown in Tables 1, 4, respectively. The list of tuned hyperparameters for the different sampling-classifier pairs that gave the best results is attached in Supplementary Tables 5A–D.

**Table 4:** Performance measure for various sampling technique and classifier pairs (Validation sets).

**Dataset identifier**	**Sampling technique—classifier**	**Sensitivity (95% CI)**	**Specificity (95% CI)**	**AUROC (95% CI)**	**MCC (95% CI)**
GSE54514	IHT-BRF	0.667 (0.662–0.672)	0.740 (0.732–0.746)	0.703 (0.691–0.708)	0.349 (0.335–0.355)
	IHT-ET	**0.704 (0.688–0.709)**	0.700 (0.687–0.712)	0.701 (0.696–0.709)	0.339 (0.330–0.345)
	IHT-BRF+ET	0.629 (0.619–0.635)	**0.800 (0.789–0.810)**	0.714 (0.709–0.718)	0.386 (0.379–0.390)
	IHT-ET+GB	0.629 (0.618–0.634)	0.790 (0.780–0.808)	0.709 (0.701–0.712)	0.374 (0.370–0.380)
	IHT-BRF+GB+ET	0.629 (0.619–0.635)	**0.800 (0.788–0.809)**	0.715 (0.710–0.720)	0.387 (0.378–0.390)
	REDN-BRF	0.670 (0.652–0.681)	0.780 (0.758–0.811)	**0.723 (0.719–0.727)**	**0.395 (0.380–0.400)**
	REDN-EE	0.629 (0.619–0.635)	0.770 (0.741–0.779)	0.700 (0.682–0.709)	0.352 (0.340–0.360)
	REDN-BRF+LOGIT	0.670 (0.652–0.682)	0.770 (0.741–0.779)	0.718 (0.710–0.721)	0.382 (0.375–0.391)
	REDN-BRF+XGBoost	0.670 (0.652–0.682)	0.740 (0.731–0.749)	0.703 (0.691–0.709)	0.350 (0.341–0.359)
E-MEXP-3850	RUS-ET (tree-based)	**1 (1.0–1.0)**	**0.913 (0.889–0.919)**	**0.956 (0.945–0.961)**	**0.828 (0.821–0.832)**
	RUS-ET (LASSO)	1 (1.0–1.0)	0.869 (0.861–0.872)	0.934 (0.929–0.941)	0.761 (0.759–0.769)
	EDN-GB	1 (1.0–1.0)	0.782 (0.779–0.789)	0.891 (0.887–0.899)	0.653 (0.649–0.659)
E-MEXP-3567	REDN-BRF	**1 (1.0–1.0)**	0.33 (0.282–0.339)	0.667 (0.652–0.671)	0.44 (0.429–0.448)
	REDN-LOGIT	0.83 (0.819–0.841)	**0.83 (0.821–0.839)**	**0.83 (0.820–0.837)**	**0.67 (0.664–0.678)**
	IHT-LOGIT	0.83 (0.821–0.839)	0.67 (0.663–0.680)	0.75 (0.744–0.76)	0.52 (0.512–0.530)
GSE40586	RUS-BRF	**0.875 (0.869–0.881)**	0.769 (0.761–0.773)	**0.822 (0.817–0.830)**	**0.626 (0.659–0.632)**
	RUS-ET	0.625 (0.619–0.632)	0.769 (0.761–0.773)	0.697 (0.691–0.701)	0.394 (0.389–0.399)
	RUS-BRF+ET	0.625 (0.619–0.633)	**0.846 (0.839–0.85)**	0.736 (0.729–0.74)	0.485 (0.479–0.49)
	IHT-BRF	0.875 (0.869–0.882)	0.692 (0.685–0.7)	0.783 (0.779–0.789)	0.551 (0.549–0.559)
	IHT-GB	0.875 (0.869–0.882)	0.691 (0.685–0.71)	0.784 (0.779–0.786)	0.551 (0.549–0.558)

*Top average values obtained for each classification metric are indicated in bold*.

### Both the Derivation and Validation Datasets Displayed Significant Distributional Differences in Terms of the Top Gene and Clinical Variables

**Figure 7:**
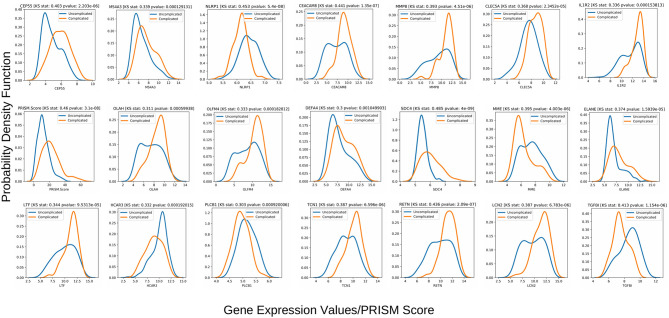
Class-wise gaussian kernel density plots for the top performing variables along with the KS test scores built using the gene expression values from the 228 patients included in the derivation dataset GSE66099. The x-axis represents the gene expression values and the y-axis represents the probability density function. A Kolmogorov-Smirnov test is a non-parametric test used to compare the equality of probability distributions. There are two scores associated with a KS test: a KS statistic that is used to quantify the distance between two distributions and the p-value which tells us the significance of the result. The differences in the distribution between the complicated and uncomplicated course groups in terms of the top 20 gene predictors and a severity score (PRISM) is shown in this plot.

The Kolmogorov-Smirnov test (or KS test) (47) is a non-parametric test of equality of two continuous distributions and is used to quantify the distance between two empirical distribution functions. The results of the two-sample KS-tests using the derivation set (GSE66099) are shown in Table 1. Both the value of the statistic and the *p*-value is given for each variable. A higher value of the statistic corresponds to greater distributional differences between the two experimental groups. For the derivation dataset (GSE66099) containing 228 patients, the Gaussian kernel density plots for the 20 genes and one clinical variable (PRISM score) demonstrating significant (*p* < 0.1) distributional differences is shown in Figure 7 and Table 1. The KS statistic values range from 0.3 to 0.485. The gene expression values of *SDC4* displayed the highest significant distributional difference (KS statistic = 0.485) between the complicated and the uncomplicated course groups. The only clinical variable (PRISM score) also displayed a significantly high KS statistic value (=0.460). From this analysis, we can clearly see that the distributions of the top gene and clinical variables have statistically significant differences between the complicated and uncomplicated course patients.

Using the same top 20 genes, we repeated the two sample KS-tests on all four validation sets (Supplementary Table 6B). Since the GSE54514 dataset had APACHE II scores, we evaluated the sensitivity of the model toward different score cutoffs of 15, 20, 25, and 30 (Supplementary Table 6A). The list of genes that showed a significant distributional difference between the two classes and their corresponding KS scores are shown in Supplementary Table 6A. It can be seen from the analysis that an APACHE II cutoff of 25 gave the highest number (10) of significant genes showing that only a fraction of the top 20 biomarkers discovered using pediatric data is actually indicative of a complex course outcome in an adult cohort. We then performed repeated 5-fold cross-validation experiments on the validation data using these 10 genes and achieved the best results using the sampling technique SMOTE and XGBoost classifier pair (mean AUROC = 0.72; mean Sensitivity = 0.78; mean Specificity = 0.65; mean MCC = 0.4). These results were similar to the external validation results for the GSE54154 dataset mentioned in the previous section. For the E-MEXP-3850 dataset, we derived a ranked list of the 20 gene variables using a tree-based variable ranking method and achieved a high AUROC of 0.956 (Table 4). After performing the two-sample KS test using this ranked list, we further achieved a list of 11 candidate gene variables (Table 1) that displayed a statistically significant distributional difference between the two classes. Repeating the external validation analysis using this set of 11 genes gave us comparable classification performances (AUROC = 0.956, Specificity = 0.913, Sensitivity = 1.0, and MCC = 0.828). For the remaining datasets, E-MEXP-3567 and GSE40586, we repeated the KS test analysis on the list of reported genes obtained using the LASSO-based variable selection technique. In both cases, we found significant distributional differences between the respective classes. The complete list of genes and the KS test results for all four validation sets are shown in Supplementary Table 6B.

## Discussion

### Functional Role of the Identified Biomarkers in Sepsis

This study revealed differentially expressed genes in peripheral blood samples from a previously published gene expression dataset collected within 24 h of PICU admission. Among the most significant genes from our machine learning analysis of complex sepsis trajectories, we found genes that are responsible for innate immune response. Sepsis is caused by the dysregulation of the host response to an infection and in its severe form, causes life-threatening organ dysfunction. Cytokines play an important role during sepsis by regulating the immune response to the infection. A variety of pro-inflammatory and anti-inflammatory mediators contribute to the inflammatory response and an imbalance between the two directly results in dysregulation. Matrix metalloproteinase-8 (*MMP8)* and Resistin (*RETN*) have been associated with the activation of pro-inflammatory cytokines TNF-α (51, 52) which in turn stimulates systemic inflammation. *MMP8* is involved in the homeostasis of the extracellular space, largely expressed by mononuclear cells and macrophages; it has been shown to be involved in roles supporting innate immunity (53). *MMP8* knockout mice have been observed to have reduced phagocytosis and NET activity (54). A number of the identified genes have been long implicated in neutrophil extracellular traps (NETs), the activation of the NET results in NETosis, which can significantly complicate disease course. Among our genes, Olfactomedin 4 (*OLFM4*), Elastase Neutrophil Expressed (*ELANE*), and lactotransferrin (*LTF*) were identified (42, 55, 56). Recent findings have suggested CEA Cell Adhesion Molecule 8 (*CEACAM8*), C-Type Lectin Domain Containing 5A (*CLEC5A*) (57) may be further implicated in NETosis. Polymorphonuclear neutrophils, typically pro-inflammatory, also perform immunoregulatory roles, by expressing *CEACAM8* and thus releasing soluble *CEACAM8* after activation. Extracellular chromatids have been shown to activate the secretion of *CEACAM8* through degranulation (58). A recent study finds *CLEC5A*, which has long been associated with dengue virus-induced lethal disease (59), to be an important factor associated with modulating innate immune response against bacterial infection in mice. This study suggests in mice with a knockout gene (CLEC5A), that prognosis was severe and by day 5, the mice had significant liver necrosis and increased risk for death. While these genes have been previously associated with mortality, in this work we show that these genes are also implicated in complex disease courses, even among survivors.

A combination of the genes identified in this analysis also has been involved in microbiome homeostasis. Specifically, Lipocalin-2 (*LCN2*), an innate immune protein has been associated with maintaining an intestinal barrier against oxidative stress, having immunosuppressive character, and protects against multi-organ dysfunction (60–62). This protein has been increasingly suggested to be a therapeutic candidate to protect against gut-origin sepsis (62).

The complexity of the disease may also contribute to the ambiguity in identifying the correct class of pathogens, specifically in gram-negative/gram-positive bacterial differentiation. Therefore, interest has emerged in differentiating these characteristics through gene expressions, and Interleukin 1 Receptor Type 2 (*IL1R2*) has been specifically implicated (63) in this. Identifying such differentiation may also aid in determining the complexity and severity of sepsis by investigating the specific types of toxins released by either class of pathogens. Superantigens, for instance, produced by *S*. *aureus* and *S*. *pyogenes* have been suggested to cause a massive cellular immune response, leading to fatal toxic shock (64), while other microbial toxins have been involved in significant sepsis-led immunosuppression (65). Hence, earlier identification of such differentiation can improve case management and therapeutic selection.

We identified Hydroxycarboxylic Acid Receptor 3 (*HCAR3*) and Membrane Metalloendopeptidase (*MME*) as two of the three downregulated genes similar to the findings of Kangelaris et al. (49) that study changes in gene expression among Acute Respiratory Distress Syndrome (ARDS) patients affected with sepsis. The genes overexpressed in ARDS were often found to be associated with a more severe sepsis outcome in other studies (including *MMP8, RETN*, and *OLFM4*) (66, 67). Similar results were found among patients with Acute Kidney Injury (AKI) where the overexpression of genes such as *MMP8, IL1R2*, and *OLFM4* was associated with increased severity and organ failure (50).

Some of the genes identified in this work overlap with approaches to predict sepsis mortality (17). DEGs identified in our study namely, *CEACAM8, IL1R2, CEP55, TGFBI*, and *DEFA4* belonged to that list. Among the PERSEVERE genes (14) that were identified as DEGs, our analysis included *MMP8, ELA2, LCN2, LTF, and RETN*, while those overlapping with the PERSEVERE XP genes (15) included *CEP55, TGFBI*, and *MME*.

Among the top consistently chosen variables (that were not DEGs) in our machine learning analysis, Syndecan-4 (*SDC4*) has been found to be a potential biomarker with an anti-inflammatory function in patients with acute pneumonia (48). Microbiologic results of the patients from our dataset, identified *Pneumococcus* as the second most frequently occurring pathogen hence this result is quite significant. We also identified Phospholipase C Beta 1 (*PLCB1*) as one of the top genes that were not a DEG. Phospholipase (*PLC*) proteins are a class of membrane-associated enzymes that hydrolyze phospholipids and are responsible for signal transduction and gene transcription. The hydrolysis of phospholipids results in the activation of protein kinase C (PKC) signaling which in turn regulates macrophage mediated inflammatory response (68). Finally, the NLR Family Pyrin Domain Containing 1 (*NLRP1*) gene, which is one of the many genes responsible for the activation of the NLR-inflammasome cascades was found to be significantly altered among septic patients in a recent study (69).

The exclusion of patients meeting SIRS criteria resulted in a lower number of DEGs, and the machine learning prediction scores were also reduced, with an AUROC of 0.61. This implies that the inclusion of such patients within the non-complicated disease course resulted in a more robust characterization of complex patients, thereby improving the predictive performance. Moreover, five genes, namely *TGFBI, DEFA4, CEP55, MME*, and *OLAH*, all except *CEP55* are implicated as early markers of neutrophil activity. *CEP55* overexpression has been implicated in T-cell lymphoma and genome instability (70, 71).

### Refining Gene Profiles Through Variable Selection and Stability Analysis

The gene expression profiles of the top 20 gene markers and one clinical variable (PRISM score) from the stability analysis had significant distributional differences between the complicated and uncomplicated course patients (Figure 7 and Table 1). When tested on a closely related independent adult validation set, two pediatric datasets and a mixed-age dataset, we achieved an out-of-sample AUC of 0.72, 0.95, 0.83, and 0.82 respectively. The relatively lower AUC in the case of the adult cohort can be attributed to the fact that the derivation cohort is based on pediatric patients (mean age 3.81 years) while this validation cohort (GSE54514) is based on adult patients (mean age 59.13 years). Some of the gene predictors that are indicative of a complex course outcome for pediatric patients, might not play the same role for adult patients. This was further justified when we found that out of the top 20 gene biomarkers, only 10 had a significant class-wise distributional difference using the gene expression profiles from the validation cohort (GSE54514) using an APACHE II cutoff of 25 (Supplementary Table 6A). In the case of the two pediatric cohorts (E-MEXP-3850 and E-MEXP-3567), where we used 28-day mortality and in-hospital mortality as class labels, respectively, the number of top genes that had a significant distributional difference between the two classes were 11 and 10, respectively. This can be attributed to the fact that unlike these two validation cohorts, the variables used for the derivation cohort were based on a complicated course outcome (See Data collection under the Materials and Methods section) and not mortality. In the derivation cohort, 28 out of the 52 complicated course patients died. Finally, for the fourth validation set, a set of 15 out of the 20 genes gave the best AUROC of 0.822. One of the consistently chosen top variables (Figure 7 and Table 4) from our analysis using the derivation data (GSE66099) was the PRISM score which could not be used for the validation analysis because this information was not available in any of the validation cohorts. Also, even within the validation datasets, the complex disease course was interpreted based on severity of illness scores that were available at the time of ICU admission or mortality. Hence, a more similar dataset which derives complex disease courses longitudinally during ICU stay may have improved the performance of the proposed biomarkers.

### Limitations

There are some limitations to our study. First, we present in this study a novel hybrid method of biomarker discovery that identifies stable and consistently chosen variables among multiple iterations of the pipeline as candidate gene markers of complex disease course. While we try to balance this novel approach by demonstrating traditional methods of identifying DEGs, further investigations in other datasets may be required to establish its validity. Second, due to the lack of any similar public dataset that identifies complicated disease courses within sepsis patients, we identified four closely related datasets which present severity of illness scores and mortality, using which we derived our interpretations of complicated and uncomplicated courses. Therefore, to ensure generalization and minimize selection bias, further validation must be performed on closely related prospective datasets. Third, in this study, we focus only on biomarkers derived from circulating blood leukocytes. Circulating biomarkers associated with cells from other dysfunctional organs, including tissue macrophages and vascular cells, might also be involved in a complicated clinical course for sepsis and were not included in this study. Finally, complicated and uncomplicated course outcomes were defined clinically, and so there may be some limitations in that interpretation, which contributes to bias.

### Rapid Phenotyping of Complex Critically Ill Patients for Improved Situational Awareness

Sepsis is a highly heterogeneous condition requiring significant clinical resources to identify, manage, and forecast disease trajectories (4). Current work in sepsis biomarker discovery is primarily centered around the use of mortality as a key indicator of outcomes, with the assumption that the disease trajectory is uniform among survivors and non-survivors. Several traditional biomarkers such as Procalcitonin (PCT) and C-reactive protein (CRP) have been extensively used to predict mortality among septic shock patients due to their high prognostic value (72, 73). While elevated levels of CRP have been associated with acute inflammation, organ failure, and mortality (74), PCT levels have been used as an indicator for decreased length of antibiotic treatments (75). However, CRP suffers from low specificity in diagnosing patients with systemic inflammation (76), and variation in early PCT levels is dependent on the type and severity of the initial infection and not necessarily on the severity of the disease itself (77). The primary outcome in most of these studies was either diagnosis of sepsis or mortality. The novelty of the proposed biomarker signature discussed here lies in identifying a holistic panel of non-invasive biomarkers to predict complicated sepsis among patients admitted to the PICU. This will minimize heterogeneity and cost in high-risk patient management.

While mortality is an important predictor variable, it does not capture the dynamic nature of several sepsis-related complications, including, but not limited to disease severity, intervention effectiveness and progressive multi-organ dysfunction. Conversely, the use of complicated course outcomes as a predictor variable is associated with poor outcomes and has been proposed as clinically relevant endpoints in several studies (78, 79). The biomarkers described in our study, which are predictive of a complicated course outcome among pediatric patients, will ultimately aid in situational awareness and clinical decision making as it pertains to the degree of care management required for patients suspected of sepsis. Unlike a set of biomarkers that may predict mortality, these collective genes enable clinicians to identify those patients who may likely survive the ICU stay but develop significant complications that result in significant expenditure of resources and clinical interventions.

## Conclusion

This paper presents a novel list of genes that predict a complicated disease course for critically ill patients in the pediatric intensive care unit. The list of 20 genes was derived from a rigorous variable selection and validation pipeline, wherein we measure variable stability over 10 simulated iterations. While these genes have been previously associated with mortality, in this work we show that these genes are also implicated in complicated course outcomes, even among survivors. Many of the derived genes were attributed to an innate immunity function and contributed to NETosis. The resulting derivation AUROC of 0.82 and validation AUROCs of 0.723, 0.956, 0.83, and 0.82 suggests that these markers can reliably predict the outcome given only a single test of peripheral blood. Finally, this is an explorative study of biomarker discovery using computational predictions derived from publicly available gene expression data. Further evaluation of the performance of these genes in discriminating complicated course outcomes will require experimental validation using closely related prospective datasets and eventually within a prospective trial.

## Data Availability Statement

The original contributions presented in the study are included in the article/Supplementary Material, further inquiries can be directed to the corresponding author/s.

## Ethics Statement

The studies involving human participants were reviewed and approved by UTHSC. Written informed consent to participate in this study was provided by the participants' legal guardian/next of kin.

## Author Contributions

RK conceived and designed the study. SB, RK, and AM developed the method, performed the data analysis, and interpretation. HW and NP interpreted the data and critically revised the article for intellectual content. All authors wrote and proofread the manuscript.

## Conflict of Interest

HW and Cincinnati Children's Hospital Medical Center hold United States patents for the PERSEVERE biomarkers and the endotyping strategy described in this manuscript. The remaining authors declare that the research was conducted in the absence of any commercial or financial relationships that could be construed as a potential conflict of interest.

## Abbreviations

AUROC: Area Under the Receiver Operating Characteristic Curve
APACHE II: Acute Physiology And Chronic Health Evaluation II
BRF: Balanced Random Forests
CCN: Cluster Centroids
DEGs: Differentially Expressed Genes
ET: Extra Trees Classifier
EE: Easy Ensemble
GB: Gradient Boosting
IHT: Instance Hardness Threshold
KEGG: Kyoto Encyclopedia of Genes and Genomes
KS: Kolmogorov-Smirnov
LASSO: Least Absolute Shrinkage and Selection Operator
LOGIT: Logistic Regression
MCC: Matthew's Correlation Coefficient
PERSEVERE: Pediatric Sepsis Biomarker Risk Model
PRISM: Pediatric Risk of Mortality Score
RFE: Recursive Feature Elimination
REDN: Repeated Edited Nearest Neighbors
SIRS: Systemic Inflammatory Response Syndrome
SMOTE: Synthetic Minority Over-sampling Technique
XGBoost: eXtreme Gradient Boosting.
