# Supplementary material for: Machine Learning Identifies Complicated Sepsis Course and Subsequent Mortality Based on 20 Genes in Peripheral Blood Immune Cells at 24 H Post-ICU Admission
Source: Front Immunol. 2021 Feb 22;12:592303. doi: 10.3389/fimmu.2021.592303 (PMC7937924; doi:10.3389/fimmu.2021.592303)
Supplement: Supplementary file 2 [file Data_Sheet_2.docx]

### **1. Preprocessing - Normalization and batch correction**

Microarray technology measures the gene expression levels of tens of thousands of genes at a time from various biological experiments. However, during those experiments, there can be many systematic variations observed in the form of noise. Some familiar sources of systematic variations include GC content, gene length, sequencing depth, etc. Normalization provides an effective way to remove such variations and ensure comparable gene expression measurements across samples and genes.

The microarray dataset, GSE66099, consisted of each probe’s raw intensities on the array in the form of CEL files. In this study, we used the Robust Multiarray Averaging (RMA) method microarray normalization. There are three major steps included with RMA normalization:

1. Background correction to generate positive intensity values.
2. Log-transformation to avoid skewness in the expression values.
3. Quantile normalization to generate comparable measurements across samples.

Below attached are visualizations that show the effect of normalization on one of the arrays using MA plots. In an MA plot, A vs. M is plotted to show the variability in expression measurements where:

$M=log_{2}(P_{int})-log_{2}(medianP_{int})$

$A=\frac{log_{2}(P_{int})+log_{2}(medianP_{int})}{2}$

Ideally, all the data points must be clustered along the M=0 line (blue line). This is mainly because of our assumption that most of the genes are not differentially expressed and that the number of up- and downregulated genes are similar. Also, the spread of the data points increases with average intensity. The red line deviates slowly from the M=0 line with increasing A. Hence, we normalize the data to address this issue. When we compare the two plots, we observe that for the “after normalization” plot, the dependence of the variation on the expression values is not as pronounced as the “before normalization” plot. In the main text, the boxplots showing the variation in the gene expression values before and after the RMA normalization are shown in Figure 2(A) and 2(B). After normalization, the expression values were aligned towards the overall mean and variance.
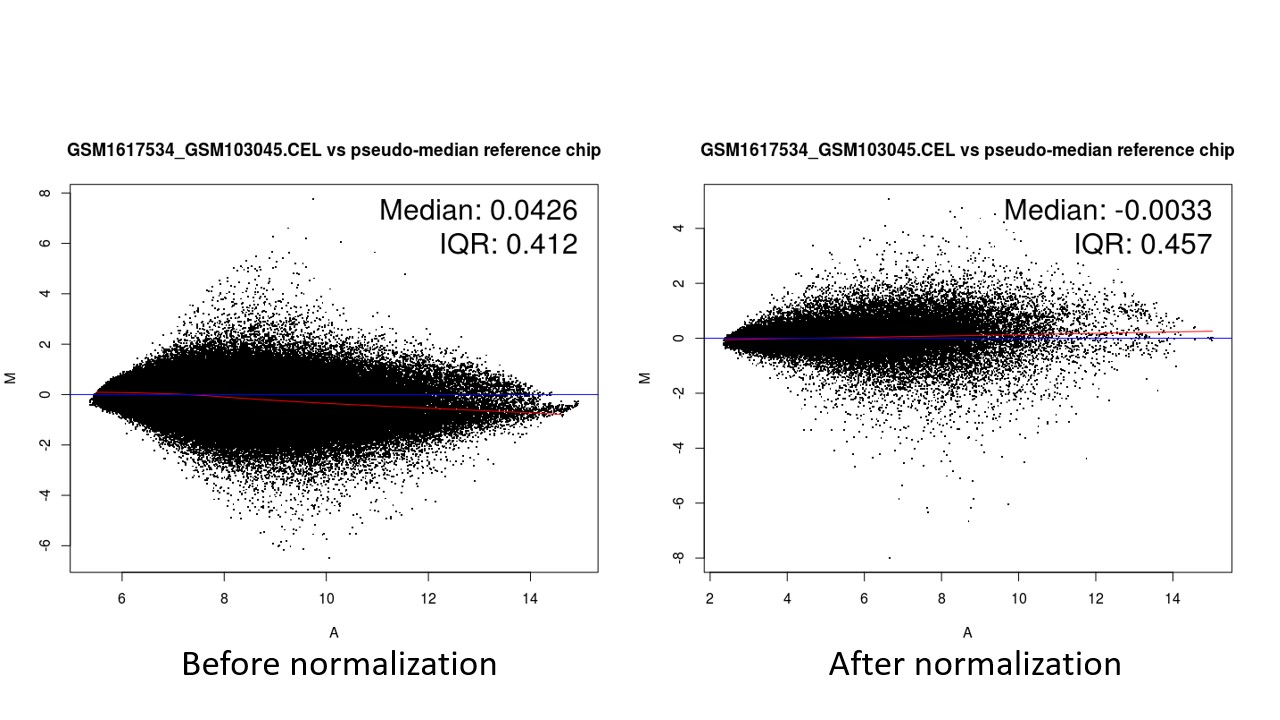


#### ***Batch Correction***

One of the most common sources of false positives in gene expression studies is batch effects. In most cases, non-biological factors from an experiment result in changes in the data that finally lead to inaccurate outcomes (e.g., list of DEGs). Our study considered the year of measurement of the gene expression data as the batch variable. Ideally, batch corrections are possible only if the batch variables are not highly correlated with the outcome (complicated course in our case).

|  | 2004 | 2005 | 2006 | 2007 | 2008 | 2010 |
| --- | --- | --- | --- | --- | --- | --- |
| Outcome= YES | 3 | 11 | 4 | 8 | 4 | 22 |
| Outcome=NO | 10 | 37 | 26 | 22 | 25 | 56 |

**Table 1: Number of gene expression measurements made for six years for the GSE66099 dataset**

From Table 1, it is clear that a tight correlation between the batch variable (year) and the outcome of interest is absent in our case. Within each batch, we have measurements from multiple different groups. So we can proceed with the batch effect removal process.

Surrogate Variable Analysis [1] is a popular method to infer batch effects and other unwanted and unmodelled sources of variation from gene expression data. The ‘sva’ package in R was used to identify batch effects in our data. Although we had prior information regarding the batch variable (the year of measurement in our case), we wanted to check if SVA could find new covariates explaining the variation in our data. The ‘sv’ component returned by the sva function contained the two new covariates or the potential batch effects. To check if the new surrogate variables (or SVs) are **associated** with the observed batch variable, a linear model is fit using the lm() in R.

| Formula | Coefficients | | | |
| --- | --- | --- | --- | --- |
|  | Components | Estimate | Std. Error | Significance level |
| Surrogate Variable 1 ~ Batch variable | Intercept | 25.84 | 3.79 | 8.6e-11 |
|  | Batch | -0.01 | 0.0019 | 8.6e-11 |
| Surrogate Variable 2 ~ Batch variable | Intercept | -0.28 | 4.16 | 0.95 |
|  | Batch | 0.0001 | 0.002 | 0.95 |

**Table 2: Results of regressing the surrogate variables returned by the sva() and the actual batch effects**

From Table 2, we can observe that the first estimated surrogate variable has a significant correlation with the batch variable. In this case, the coefficient tells us that by changing the batch variable, the value of the SV changes by -0.01, and this result is significant. So this shows that the estimated SV is associated with the batch. The boxplots displayed in Figure 2C in the manuscript also show a relationship between the inferred batch effect and the observed batch effect. This is similar to performing an ANOVA test and checking if the values of the SVs’ calculated for each sample are different between the batches (or difference in the means between the boxplots).

The resulting variation is removed using the Combat function included in the ‘sva’ package. Combat() removes batch effects using an empirical Bayesian framework [2]. To do that, we create two model matrices with the model.matrix() - the “null model” and the “full model”. The null model contains the known variables (plus covariates needed for adjustment), and the full model contains all the variables from the null model and the outcome of interest. We pass the full model (without any batch variable) and the batch variable as separate arguments to the Combat(). The output consists of a corrected expression set with the batch effects removed completely.

### **2. Machine learning workflow**

The workflow adopted for our machine learning analysis is shown in Figure 1 (main text). The entire process can be subdivided into three main parts. Here, we will discuss each part in detail:

***Part A: Cross-Validation***

A typical machine learning workflow involves dividing the available data into three groups: Train, Validation, and Test. The sample of the data that is used to fit the model is referred to as the train set. The Validation set is used to tune the hyperparameters of the model and is used to derive the best model configuration. The test set is used to provide an unbiased evaluation of the best model derived from the training and the validation set.


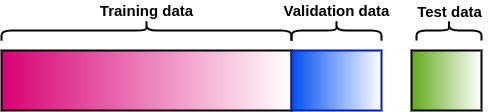


**Figure 2: A typical machine learning workflow requires splitting the dataset into three groups. (Source: Google Images)**

Whenever we are provided with a limited data sample, we train and evaluate our models using an approach known as Cross-Validation. *K-*fold cross-validation requires a single parameter *k,* which refers to the number of groups the given data sample is split into. In our case, we chose k=5. The general procedure to derive the cross-validation results is as follows:

1. Randomly shuffle the data
2. Split the dataset into 5 equal-sized subsets
3. For each subset,
   1. Consider one subset as a hold-out or a test set
   2. Take the remaining four subsets as a single training set
   3. Derive the best set of hyperparameters and train the model using the training set and test it on the test set.
   4. Calculate the evaluation metrics such as Sensitivity, Specificity, MCC, and AUC.
4. The final results are the average classification metrics calculated across all five folds.

***Stratified k-fold cross-validation***

The derivation set (GSE66099) used to identify the set of candidate biomarkers had 176 patients with an uncomplicated course and 52 patients with a complicated course outcome. Due to the skewed class distribution, we used Stratified *k*-fold cross-validation instead of normal *k*-fold. The class distribution of the dataset was preserved in each of the train-test splits.

***Repeated Cross-Validation***

A single run of the 5-fold stratified cross-validation procedure might lead to a noisy estimate of the model. In a typical 5-fold stratified cross-validation process, the first two steps include randomly shuffling the data and splitting the dataset into 5 equal-sized subsets keeping the class distribution intact in the given train-test split. This would mean that every time we run the cross-validation experiments, we use a different 5-fold split of the original data, thereby resulting in a different mean estimate of the classifier’s performance.

Repeated cross-validation is often used to improve the performance of a classifier by repeating the *k*-fold cross-validation process several times wherein each repetition, the folds are split in a different way. The mean performance is reported across all folds and all repeats.

In our analysis, we repeated the 5-fold stratified cross-validation procedure 10 times and reported the mean performance calculated across 50 different train-test experiments.

We used the RepeatedStratifiedKFold function from the model_selection module in scikit-learn [10] to perform our cross-validation experiments.

**Part B: High dimensional feature reduction**

**Scaling:** We scaled our features (genes) using a popularly used normalization technique known as Min-Max scaling. All the feature values were shifted and scaled so that ended up in the 0-1 range.

Here’s the formula for Min-Max scaling:

$\underline{X}=\frac{X-X_{min}}{X_{max}-X_{min}}$

X_max_ and X_min_ are the maximum and minimum values of a given feature respectively.

**Feature Selection I:** Gene expression data is usually high dimensional (contains measurements from thousands of genes) and highly redundant. Only a fraction of the genes are actually responsible for the outcome of interest. Dimensionality reduction is a necessary step to reduce the redundancy and dimension of gene expression data. We used four feature reduction techniques on our scaled derivation dataset.

1. **LASSO:** The Least Absolute Shrinkage and Selection Operator [11] is a powerful method that performs both regularization and feature selection at the same time.

A linear regression model can be expressed as follows:

$Y_{i}=\beta_{0}+x_{i1}\beta_{1}+...+x_{ik}\beta_{k}+\varepsilon_{i}$, $where i=1...n$

where *Y_i_* is the response variable, the parameters $\beta_{0},\beta_{1},...\beta_{k}$are the regression coefficients and we have *k* number of explanatory variables. The random error or $\varepsilon_{i}$ is assumed to have 0 mean and constant variance. Assuming *n* samples in total, the vector notation used to represent the above formula is : $Y=X\beta+\varepsilon$, where *Y* is the (n by 1) response vector, X is the (n by k) design matrix representing the k features, $\beta$is the (k by 1) coefficient vector and ɛ is the (n by 1) error vector.

The main goal of linear regression is to fit a straight line to a number of points minimizing the squared residuals. LASSO minimizes the sum of squared residuals while placing an upper bound on the model parameters’ absolute sum. Using the formulation used by Buhlmann and van de Geer [3], we get:

$minimize(\frac{{|{|Y-X\beta||}_{2}^{2}}}{n}) subject to \sum_{j=1}^{k} ||\beta||_{1}<t$

where t is the upper bound for the sum of the coefficients. This is equivalent to solving

$\hat{\beta}(\lambda)=argmin_{\beta}(\frac{{|{|Y-X\beta||}_{2}^{2}}}{n})+\lambda||\beta||_{1})$ $where ||Y-X\beta||{}_{2}^{2}=\sum_{i=1}^{n} (Y_{i}-(X{\beta)}_{i}){}^{2},||\beta||_{1}=\sum_{j=1}^{k} |\beta_{j}|$

and λ is the shrinkage parameter that controls the amount of penalty that must be applied to the $\beta$’s. When we solve this optimization problem, some of the coefficients are shrunk to zero and as a result, the features corresponding to those coefficients are excluded from the model.

This makes LASSO a powerful feature selection technique. We implemented the LassoCV function from the linear_model module for feature selection purposes. This particular function uses cross-validation to choose the best model and we used the default 5-fold cross-validation splitting strategy.

1. **mRMR:** Minimum Redundancy Maximum Relevance [4] is a feature selection algorithm to find a small subset of features by considering both the correlations between the features and their importance. If two highly correlated features are also highly relevant, then adding both of them would increase the model complexity. So for a set of S features, the relevance between them is defined as, and the redundancy is denoted by $R=\frac{1}{|S|{}^{2}}\sum_{x_{i},x_{j}\epsilon S} I(x_{i},x_{j})$ , where $I$ is the mutual information operator. The mRMR score for the given set S is given by (D-R). The goal is to find the subset of features S with the maximum (D-R). We used the Python wrapper named “pymrmr” that was published with the original paper and selected the top 10 important features using this method.
2. **Random Forests for feature selection:** Random forests [5] are made up of several decision trees trained on a random subset of observations using a random subset of features. No single tree sees all the features or all the samples at once and this makes it less prone to overfitting. Each tree, in turn, is a series of yes/no questions based on a combination of features. At each question (or node), the tree divides into two branches each of them containing samples that are more similar to one another and different from the ones in the other branch. So the importance of each feature is based on how “pure” (containing samples belonging to a single class) each of the branches is. We used the RandomForestClassifier function from the ensemble module and a collection of 100 estimators to derive the feature importances. We finally selected the top 10 ranked features for our feature pool.

We added the DEGs identified from our previous analysis to the set of genes chosen by each of the above three feature selection strategies. This formed our pooled list of features which was then passed onto the next stage of feature selection.

**Feature Selection II (Recursive Feature Elimination):** Our main goal is to identify a small subset of features to remove redundancy and avoid overfitting. This final feature selection approach tries to remove any redundant features from the pooled feature set by recursively removing them and building a model on those that remain. This process is also known as Recursive Feature Elimination [12]. This ensures that our final set of features obtained after this stage contributes most to the output.

The REFCV function from the feature selection module was used to implement this final feature selection strategy. For each of the classifiers implemented in our study, the RFECV function was called with a 3-fold cross-validation splitting strategy, and a “roc_auc” method of scoring was used as the function parameters.

**Part C:** **Model fitting**

After finding the optimal set of features from the high-dimensional gene expression data, the next step was to use these features to train our model. Hyperparameter tuning is a very crucial step to find the best set of parameters for a given classifier. For a given classifier, grid search uses an exhaustive search and evaluation strategy to achieve this objective. It checks for every single combination of hyperparameters in the grid, evaluates the model based on some predefined metric, and outputs the combination that gave the best results. It is a bit computationally expensive, especially if one uses a cross-validated grid search technique to search for the optimal parameters in a parameter grid. The GridSearchCV function from the model_selection module with the default 3-fold cross-validation strategy and a “roc_auc” scoring metric was used to search for the best set of hyperparameters.

The final classifier with the best set of hyperparameters was then trained on the subset of features derived from our rigorous feature selection strategy. This model was then evaluated on the test data set aside for evaluation at the beginning of each run of the cross-validation experiment.

***Derivation of the final set of stable features (genes) from the cross-validation experiments***

All the steps of the machine learning workflow discussed up to this point are based on a single run of the 5-fold cross-validation experiment. We repeated this entire process ten times, choosing a different 5-fold split every time. Hence, we had 50 different sets of highly relevant features that were predictive of a complicated course outcome. The fraction of times a particular feature was chosen out of the 50 runs constituted the “Normalized Score” in Table 3. For instance, the normalized score for the gene *OLAH* was 84, meaning that it was chosen as one of the top relevant features, 84% or 42 out of 50 times.

***A general framework explaining tree-based classification and logistic regression***

In our study, we generated binary classification models using tree-based classifiers and logistic regression. The best results from our derivation dataset used to derive the 20 gene biomarkers were obtained using random forests [5]. In this section, we will discuss briefly how each of these classifiers work in the context of gene expression data.

**Random Forests - Training**

Let us consider a toy example of a gene expression study where we have measured the expression levels of four genes A, B, C and D from four samples. We already know the outcome (complicated course - yes/no) for each of those samples included in our training data.

| **Samples** | **A** | **B** | **C** | **D** | **Complicated Course (Yes or No)** |
| --- | --- | --- | --- | --- | --- |
| 1 | 5.76 | 5.34 | 5.10 | 5.2 | No |
| 2 | 6.12 | 4.51 | 6.78 | 5.56 | Yes |
| 3 | 5.19 | 6.19 | 5.10 | 4.5 | No |
| 4 | 5.26 | 4.98 | 6.12 | 6.5 | Yes |

Now the following steps will explain how to create the random forest model.

**Step 1:** Create a bootstrapped dataset that is the same size as the original by randomly picking samples with replacement.

| **Samples** | **A** | **B** | **C** | **D** | **Complicated Course (Yes or No)** |
| --- | --- | --- | --- | --- | --- |
| 2 | 6.12 | 4.51 | 6.78 | 5.56 | Yes |
| 3 | 5.19 | 6.19 | 5.10 | 4.5 | No |
| 4 | 5.26 | 4.98 | 6.12 | 6.5 | Yes |
| 4 | 5.26 | 4.98 | 6.12 | 6.5 | Yes |

**Step 2:** Create a decision tree using the bootstrapped dataset and a random subset of features (or genes). First, in this example we will randomly select two features (B and C) as candidates for the root node. Just for the sake of this example, let's consider that gene B did a good job at separating the two classes. So let we consider B as the root node.
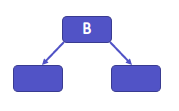


Now, we have to figure out how to split samples at the left child node. Just as we had done before, we will randomly pick two genes out of the remaining genes (A,C and D) as candidates for the left child node. We continue building the tree in this fashion but considering only a random subset of features at each step. The following tree is created as a result and we name this as Tree 1.


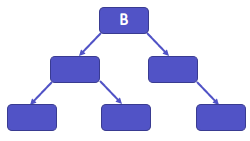
Now we repeat the entire process of creating trees on bootstrapped samples of the data using a random subset of variables at each step. Finally we get a collection of trees using the training data as shown below.


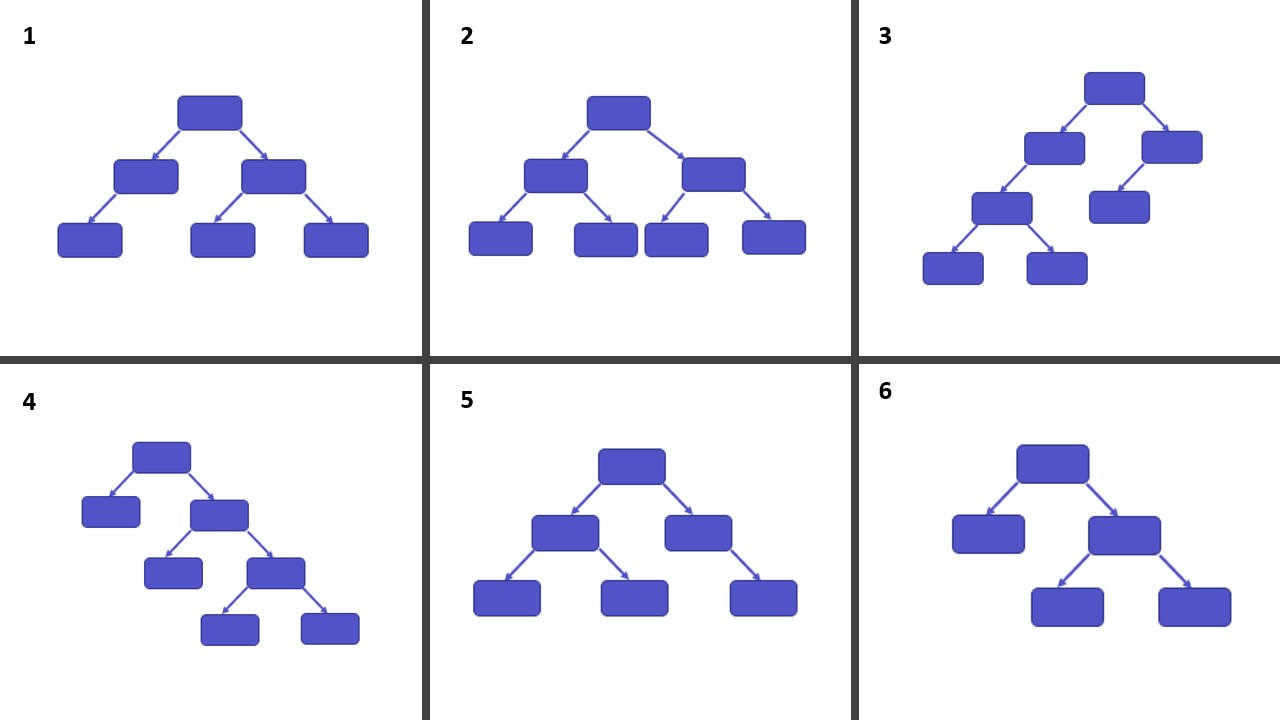


Ideally we will have hundreds of trees built using bootstrapped samples of the original data considering only a subset of the variables at each step. Due to space constraints, we are showing only six.

**Random Forests - classifying a new sample using the collection of trees**

Now let’s say we have a new sample as follows and we want to check whether this patient will follow a complicated course outcome.

| **A** | **B** | **C** | **D** | **Label** |
| --- | --- | --- | --- | --- |
| 6.76 | 5.25 | 4.55 | 5.19 | ?? |

We take this data and run it down the first tree and obtain a prediction. We continue this process and obtain predictions using trees 2,3,4,5 and 6. Below are the results we obtain for instance,

| **Tree** | **Predictions** |
| --- | --- |
| 1 | Yes |
| 2 | Yes |
| 3 | Yes |
| 4 | No |
| 5 | Yes |
| 6 | Yes |

We see that the majority vote in this case is “Yes”. Hence, we conclude that this new test sample will follow a complicated course outcome.

**Random Forests - evaluation**

When we created the bootstrapped dataset in step 1, we allowed duplicate entries (sampling with replacement) and as a result we left out sample 1 while creating the first tree. (Note: In reality, this would be a much larger dataset instead of just one sample). This is also known as the “**Out-of-Bag Dataset**” for Tree 1. We evaluate Tree 1 using this sample and check if we get the correct prediction, i.e.,”No”. We repeat this for all 6 trees and check if each of them correctly classifies their out-of-bag samples. The proportion of the out-of-bag samples that were incorrectly classified is known as the **“Out-of-Bag Error”.** Now that we have a method to evaluate the accuracy of the random forests, we repeat the entire process by choosing different initial settings, such as considering three instead of two features at random to build the tree at each step. In this way we choose the most accurate random forest.

Now we will explain various types of tree-based classifiers used in our study in the context of imbalanced classification

**Balanced Random Forest classifier:** This is a variant of random forest designed to work with imbalanced data [6]. Basically, it performs random undersampling of the majority class for each bootstrapped sample. This is done to explicitly change the class distribution of the samples while training.

**Easy Ensemble classifier:** Only a subset of the majority class and all examples from the minority class is chosen while training an easy ensemble classifier [7]. Also, an easy ensemble classifier uses boosted decision trees instead of pruned decision trees. This method performs well in general when we have high class imbalance and need to produce a model that is generalizable in practice.

**Extra trees classifier:** The extra trees classifier [9] is very similar to random forests and differs in only two major aspects:

1. Instead of performing bootstrap sampling with replacement, it uses the whole original data.
2. While choosing the selection of points to split the nodes, random forests always try to find the optimal split which gives the least out-of-bag error but extra trees choose the split randomly.

**Gradient Boosting:** Gradient boosting [8] is an ensemble technique used to combine several weak classifiers in order to get better classification power. There are three main components that define a gradient boosting classifier:

1. A loss function to be optimized. For classification, this might be logarithmic loss.
2. Decision trees are used as weak learners and are constructed in a greedy manner.
3. Decision trees are added one at a time and a gradient descent procedure is used to reduce the loss at each step of process. In an effort to improve the final output of the model the output of a new tree is added to the existing output obtained from a sequence of trees.

This process is continued until we have achieved an acceptable classification error on the external validation data.

**Logistic Regression:** In the logistic model, the log-odds for the binary label is a linear combination of one or more independent variables or features. Our training data has four genes (A,B,C and D) and a binary outcome variable (1=complicated course, 0=uncomplicated course) which we denote $p=P(Y=1)$*.* We assume a linear relationship between the genes and the log-odds of the event that $Y=1$. This can be represented as,

$l=log{}_{b}\frac{p}{1-p}=\beta_{0}+\beta_{1}A+\beta_{2}B+\beta_{3}C+\beta_{4}D$

Recovering the odds by exponentiating the log-odds, we get

$\frac{p}{1-p}=b{}^{\beta_{0}+\beta_{1}A+\beta_{2}B+\beta_{3}C+\beta_{4}D}$

$Finally, P(Y=1)=p=S_{b}(\beta_{0}+\beta_{1}A+\beta_{2}B+\beta_{3}C+\beta_{4}D)$

where S_b_ is the sigmoid function with base b.

From the above equation it is clear that if we can estimate the parameters or the $\beta_{i}'s$, then we can find the probability that Y=1 for a given set of gene expression values.

***Distribution plots***

A distribution plot displays the variation in the data distribution. A Kernel Density Plot is a type of distribution plot that estimates the continuous probability density function of a random variable and produces a smooth curve as an output.

The figure below shows the KDE plot of two normal continuous variables with different means.

**
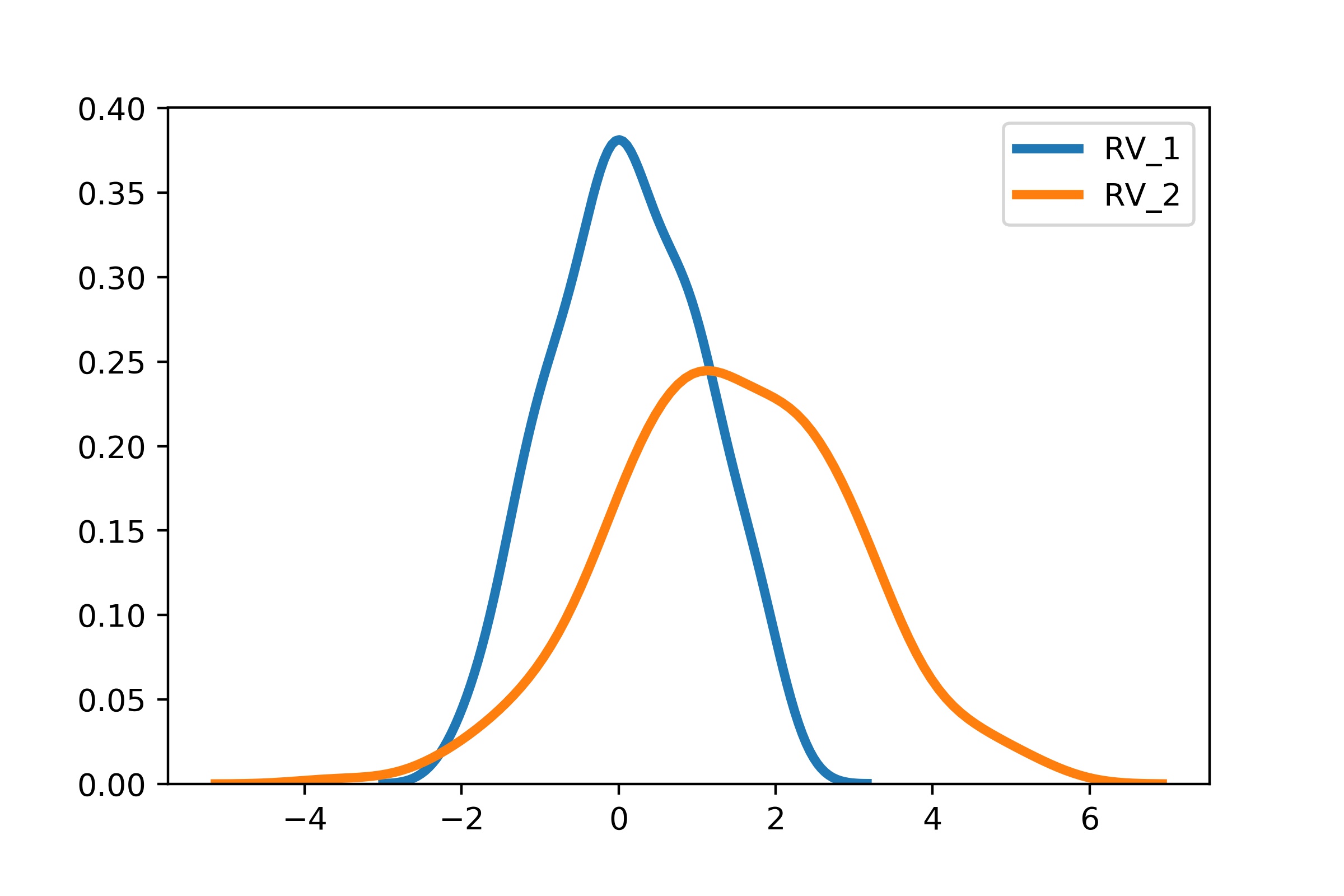
Figure 3: KDE plot showing the differences between two continuous normal random variables**

To understand the difference between the two distributions, we can perform the non-parametric Kolmogorov-Smirnov test (or KS test) [13] of equality of continuous 1-D probability distributions.

The formulation of the KS test statistic is discussed below.

***Cumulative and Empirical Distribution function***

The cumulative distribution function *F(x)* of a random variable *X* is given by,

$F(x)=P(X\leq x)$

The CDF of a random variable *X* uniquely characterizes the probability distribution.

Given observations $x_{1},x_{2},...x_{n}$*,* the empirical distribution function $F_{obs}(x)$*,* is the fraction of data points that lies below *x*,

$F_{obs}(x)=\frac{\#observations below x}{\#observations}$

If we order the observations $y_{1}\leq y_{2}\leq...\leq y_{n}$, then $F_{obs}(y_{i})=\frac{i}{n}$

Now, we want to compare the empirical distribution function, *F_obs_(x)* with the cumulative distribution function under the null hypothesis (*F_exp_*).

The Kolmogorov-Smirnov statistic is given by $D_{n}=max_{x}|F_{exp}(x)-F_{obs}(x)|$

A two-sample KS test is used to check whether the two samples are drawn from the same distribution. If the KS statistic is small or the two-tailed p value is high, then we cannot reject the null hypothesis that the two samples are drawn from the same distribution.

For the above two normal random variables with different means, performing the KS test gives us a KS statistic value of 0.438 and a p value of $2.2\times10{}^{-16}$. So we can say that there is a statistically significant difference between the two underlying probability distributions.

For each of the 20 marker genes, we demonstrate the difference in distribution between the complicated and uncomplicated course patients using Figure 7 (in the main manuscript). For a given gene, say MMP8, first we extract the expression values for each class (complicated and uncomplicated) and then compare their distributions through KDE plots as demonstrated in Figure 3 above. The KS statistic as well as the p values are reported for every gene.

**References:**

1. Leek JT, Johnson WE, Parker HS, Jaffe AE, Storey JD. The sva package for removing batch effects and other unwanted variation in high-throughput experiments. *Bioinformatics*. 2012;28(6):882-883. doi:10.1093/bioinformatics/bts034
2. Johnson WE, Li C, Rabinovic A. Adjusting batch effects in microarray expression data using empirical Bayes methods. Biostatistics. 2007 Jan;8(1):118-27. doi: 10.1093/biostatistics/kxj037. Epub 2006 Apr 21. PMID: 16632515.
3. P.B¨uhlmann, S.van de Geer: Statistics for High-Dimensional Data: Methods, Theory and Applications. Springer 2011
4. Hanchuan Peng, Fuhui Long and C. Ding, "Feature selection based on mutual information criteria of max-dependency, max-relevance, and min-redundancy," in IEEE Transactions on Pattern Analysis and Machine Intelligence, vol. 27, no. 8, pp. 1226-1238, Aug. 2005, doi: 10.1109/TPAMI.2005.159.
5. Breiman L. Random forests. Machine learning. 2001 Oct 1;45(1):5-32.
6. Chen C, Liaw A, Breiman L. Using random forest to learn imbalanced data. University of California, Berkeley. 2004 Jul;110(1-12):24.
7. Liu XY, Wu J, Zhou ZH. Exploratory undersampling for class-imbalance learning. IEEE Transactions on Systems, Man, and Cybernetics, Part B (Cybernetics). 2008 Dec 16;39(2):539-50.
8. [Friedman JH. Greedy function approximation: a gradient boosting machine. *Annals of statistics* (2001)1189–1232.](https://www.zotero.org/google-docs/?96jfwO)
9. P. Geurts, D. Ernst., and L. Wehenkel, “Extremely randomized trees”, Machine Learning, 63(1), 3-42, 2006.
10. Pedregosa F, Varoquaux G, Gramfort A, Michel V, Thirion B, Grisel O, Blondel M, Prettenhofer P, Weiss R, Dubourg V, Vanderplas J. Scikit-learn: Machine learning in Python. the Journal of machine Learning research. 2011 Nov 1;12:2825-30.
11. Tibshirani R. Regression shrinkage and selection via the lasso. Journal of the Royal Statistical Society: Series B (Methodological). 1996 Jan;58(1):267-88.
12. Gregorutti B, Michel B, Saint-Pierre P. Correlation and variable importance in random forests. Statistics and Computing. 2017 May 1;27(3):659-78.
13. Massey Jr FJ. The Kolmogorov-Smirnov test for goodness of fit. Journal of the American statistical Association. 1951 Mar 1;46(253):68-78.
